# ISG15 as a prognostic biomarker in solitary fibrous tumour

**DOI:** 10.1007/s00018-022-04454-4

**Published:** 2022-07-21

**Authors:** Jose L. Mondaza-Hernandez, David S. Moura, María Lopez-Alvarez, Paloma Sanchez-Bustos, Elena Blanco-Alcaina, Carolina Castilla-Ramirez, Paola Collini, Jose Merino-Garcia, Jorge Zamora, Jaime Carrillo-Garcia, Roberta Maestro, Nadia Hindi, Jesus Garcia-Foncillas, Javier Martin-Broto

**Affiliations:** 1 Health Research Institute Fundacion Jimenez Diaz, Universidad Autonoma de Madrid (IIS/FJD-UAM), Madrid, Spain; 2 University Hospital General de Villalba, Madrid, Spain; 3 Institute of Biomedicine of Sevilla (IBIS, HUVR, CSIC Universidad de Sevilla), Seville, Spain; 4 CIBERONC, Instituto de Salud Carlos III, Madrid, Spain; 5 Pathology Department, University Hospital Virgen del Rocio, Seville, Spain; 6 Soft Tissue and Bone Pathology, Histopathology and Pediatric Pathology Unit, Diagnostic Pathology and Laboratory Medicine Department, Fondazione Istituto di Ricovero e Cura a Carattere Scientifico (IRCCS), Instituto Nazionale Tumori, Milan, Italy; 7 Pathology Department, University Hospital Fundacion Jimenez Diaz, Universidad Autonoma, Av. Reyes Catolicos 2, 28040 Madrid, Spain; 8 Unit of Oncogenetics and Functional Oncogenomics, Centro di Riferimento Oncologico di Aviano (CRO Aviano), IRCCS National Cancer Institute, Aviano, Italy; 9 Medical Oncology Department, University Hospital Fundación Jimenez Diaz, Madrid, Spain

**Keywords:** ISG15, Solitary fibrous tumour, Biomarker, Cancer stem cell, Drug resistance

## Abstract

**Background:**

Solitary fibrous tumour (SFT) is a rare mesenchymal malignancy that lacks robust prognostic and predictive biomarkers. Interferon-stimulated gene 15 (ISG15) is a ubiquitin-like modifier, associated with tumour progression, and with poor survival of SFT patients, as previous published by our group. Here, we describe the role of ISG15 in the biology of this rare tumour.

**Methods:**

ISG15 expression was assessed by immunohistochemistry in tissue microarrays from SFT patients and tested for correlation with progression-free survival and overall survival (OS). The effects of ISG15 knockdown or induction were investigated for cancer stem cell (CSC) characteristics and for drug sensitivity in unique in vitro models of SFT.

**Results:**

The prognostic value of ISG15 for OS was validated at protein level in malignant SFT patients, prospectively treated with pazopanib and enrolled in GEIS-32 trial. In SFT in vitro models, ISG15 knockdown lead to a decrease in the expression of CSC-related genes, including *SOX2, NANOG, ALDH1A1, ABCB1 and ABCC1*. Likewise, ISG15 downregulation decreased the clonogenic/ tumoursphere-forming ability of SFT cells, while enhancing apoptotic cell death after doxorubicin, pazopanib or trabectedin treatment in 3D cell cultures. The regulation of CSC-related genes by ISG15 was confirmed after inducing its expression with interferon-β1; ISG15 induction upregulated 1.28- to 451-fold the expression of CSC-associated genes.

**Conclusions:**

ISG15 is a prognostic factor in malignant SFT, regulating the expression of CSC-related genes and CSCs maintenance. Our results suggest that ISG15 could be a novel therapeutic target in SFT, which could improve the efficacy of the currently available treatments.

**Supplementary Information:**

The online version contains supplementary material available at 10.1007/s00018-022-04454-4.

## Background

Solitary fibrous tumour (SFT) is a rare type of soft-tissue sarcoma (STS), with an estimation of 1 case per million people each year. These tumours were firstly described in the pleura, and they can appear anywhere in the body. In general, SFT consists of a well-encapsulated fibroblastic body that presents a significant collagenous component and prominent branching staghorn vasculature [1]. It is characterized by *NAB2-STAT6* gene fusion, which is believed to be key for tumorigenesis [2–4]. This mesenchymal neoplasm can manifest in various clinically and histologically different subtypes. On histology, typical and malignant SFT (T-SFT and M-SFT respectively) are distinguished based on the mitotic index and/or presence of necrosis. Of note, dedifferentiated M-SFT (DD-SFT) is an extremely aggressive subtype, which presents an abrupt transition into a high-grade sarcoma [1, 5]. However, these histologic features hardly predict SFT clinical course. Therefore, the most recent WHO classification suggests the use of risk-stratification models. The 3-variable risk model takes age at diagnosis, tumour size and mitotic index into account, while the 4-variable model includes also necrosis [1].

Localized T-SFT is commonly indolent, with surgical resection being the most effective treatment. However, very limited therapeutic options are available for advanced disease, when surgical intervention is impracticable. What is more, the monitoring of advanced cases is hindered due to the lack of efficient prognostic and/or predictive biomarkers in SFT. Recently, a phase-II clinical trial carried out by the Spanish Group for Sarcoma Research (GEIS), in collaboration with the French (FGS) and Italian (ISG) sarcoma groups, showed promising activity of antiangiogenics (i.e. pazopanib) in both T-SFT and M-SFT [6, 7]. Remarkably, in the M-SFT cohort, *ISG15* proved to be a relevant prognostic factor for progression-free survival (PFS) and overall survival (OS), both in the univariate and multivariate analysis.

ISG15 is a 15 kDa ubiquitin-like protein that can be secreted to the extracellular medium, found intracellularly in its free form, or can be covalently bound to other target proteins, in a process known as ISGylation [8–11]. ISGylation was at first studied for its role in the antiviral immune response. Following viral infection, type-I interferons (IFNα/β) are known to induce ISG15 expression and its conjugation with target proteins [12]. Recent studies have focused on the role of ISG15 in other major cellular processes, such as DNA repair [13–15], autophagy [16, 17] or protein translation, as well as in pathological contexts like genotoxic stress or tumour development [18–25]. In sarcoma patients, *ISG15* expression is up-regulated in tumour tissue compared to normal tissue, and its expression was included in a metastasis-related genetic signature for poor prognosis [26]. In addition, ISG15 and ISGylation have been associated with cancer stem cell (CSC) maintenance and behaviour, which can be translated into poor prognosis in patients. Of note, in pancreatic ductal adenocarcinoma (PDAC), not only does ISG15 extracellular paracrine-signalling play a key role in CSC maintenance [27, 28], but also the intracellular ISG15 and ISGylation are required for CSC metabolic plasticity and mitophagy [29]. Besides, ISG15 can be a crucial microenvironmental factor in this malignancy, as tumour-associated macrophages can secrete ISG15, increasing CSC phenotype in tumour cells [30].

On these grounds, we addressed the prognostic value of ISG15 in a well characterized cohort of SFT. In parallel, the role of ISG15 in the maintenance of the CSC-like phenotype and drug resistance was investigated in SFT pre-clinical models.

## Methods

### Patients and samples

Study samples were prospectively collected within the GEIS-32 clinical trial (ClinicalTrials.gov number NCT02066285 and European Union Drug Regulating Authorities Clinical Trials (EUDRACT), number 2013-005456-15). Patient inclusion, treatment, monitoring, and endpoint criteria were described in previously published articles [6, 7], as well as sample processing and next-generation sequencing. Statistical analysis was performed on a total of 49 patients, from which samples for either RNA (*n* = 45) or protein (*n* = 43) were available. Non-evaluable samples from each group correspond to lack of biological material.

### Immunohistochemistry and tissue microarray (TMA) constructs

Two representative areas (1 mm in diameter) per tumour were selected, based on haematoxylin/eosin staining, for the generation of a tissue microarray (TMA). A TMA instrument (Beecher Instruments; Sun Prairie, WI, USA) was used for TMA assembly. Immunohistochemistry was performed in TMAs 4-µm sections, using an anti-ISG15 monoclonal antibody (sc-166755, Santa Cruz Biotechnology, Dallas, TX, USA). The percentage of ISG15-positive tumour cells was evaluated using the following scoring system: negative (0% positive cells), low (+ , 5–25% positive cells), intermediate (+ +, 25–50% positive cells) and high (+ +  + , > 50% positive cells). Staining intensity of ISG15 was graded as negative, weak, or strong. Samples with a negative/low percentage of ISG15-positive cells or negative/weak intensity were included in the low ISG15 group. Samples with an intermediate/high percentage of positive cells or strong intensity were included within high ISG15 group. ISG15 staining was independently evaluated by two sarcoma expert pathologists, blinded for clinical data. Normal liver tissue was used as a positive control for ISG15 staining, according to the manufacturer’s instructions. ISG15 expression levels were quantified by HTG EdgeSeq technology as described before [6, 7]. Upper quartile Q3 was considered the cut-off value to discriminate between high and low ISG15 expression groups.

### Statistical analysis

Overall survival (OS) and progression-free survival (PFS) were measured from the date of initial treatment, within the clinical trial, to the final event (patient death for OS, disease progression according to Choi criteria or death for PFS) and were estimated according to the Kaplan–Meier method. The associations between the variables of interest (i.e., protein/gene expression and clinical outcomes) were performed by the log-rank test, statistical significance was defined at *p* = 0.05. Hazard ratios (HR) for the multivariate analysis were calculated following Cox’s regression. All the statistical procedures were performed with SPSS 22.0 software (IBM, Armonk, NY, USA).

### In vitro experiments

#### Cell lines and culture conditions

Malignant SFT stabilised cell line INT-SFT (established in the Maestro lab by SV40 LargeT Antigen-mediated immortalization) [31], malignant SFT primary cell line IEC139 (established at the Martin-Broto lab), liposarcoma cell line 93T449 (ATCC^®^ CRL-3043™; ATCC, Old Town Manassas, VA, USA), leiomyosarcoma primary cell lines AA (kindly provided by Dr Amancio Carnero of the Institute of Biomedicine of Seville, CSIC, US, HUVR; Seville, Spain) and CP0024 (established at the Martin-Broto lab), angiosarcoma primary cell line ICP059 (established at the Martin-Broto lab), malignant peripheral nerve sheath tumour (MPNST) primary cell line ICP060 (established at the Martin-Broto lab), SW982 (ATCC^®^ HTB-93™; ATCC) synovial sarcoma cell line, fibrosarcoma cell line HT-1080 (ATCC^®^ CCL-121™; ATCC) and HEK293T cells (ATCC^®^ CRL-3216™; ATCC) were used for this study. INT-SFT, 93T449, CP0024, ICP059 and ICP060 cell lines were cultured in RPMI medium (Gibco™, Thermo Fischer Scientific, Waltham, MA, USA), HT-1080 and AA cell lines were maintained in F-10 medium (Gibco™), SW982 cell line was cultured in Leibovitz’s L-15 Medium (Gibco™) and HEK293T cell line in DMEM medium (Gibco™). All cell culture mediums were supplemented with 10% FBS (Gibco™), and 100 units/mL penicillin and 100 µg/mL streptomycin (Sigma-Aldrich, San Luis, MO, USA). Cells cultures were kept at 37 ºC in a 5% CO_2_ atmosphere and tested routinely for mycoplasma or fungi contamination. All cell lines were discarded after 2 months, and new lines obtained from frozen stocks.

#### ISG15 in vitro silencing

HEK293T cells were transfected by the calcium phosphate method, with lentivirus-producing plasmids PMD2.G-VSV-G and pCMV-dR8.91 (Addgene, Watertown, MA, USA); and the plkO.1-puro plasmid containing the sequence for either non-targeting (SHC016-1EA, Sigma-Aldrich) or ISG15 shRNA (TRCN0000007421, Sigma-Aldrich). After 24 h medium was replaced with fresh DMEM. 48 h-post transfection the lentivirus-containing media were filtered through a 0.45 µm syringe filter, supplemented with 4 mg/ml polybrene (Sigma-Aldrich) and used to transduce INT-SFT or IEC139 cells. Two additional cycles of infection were carried out every 12 h. Transduced cells were selected using 0.5 µg/ml puromycin (Thermo Fisher Scientific) for 5–7 days. For long-lasting silencing, clonogenic cell lines were established through single-cell sorting using BD FACSJazz (BD Biosciences, Franklin Lakes, NJ, USA). Silencing was verified by western blot (WB) and real-time quantitative reverse polymerase chain reaction (RT-qPCR) analysis.

#### Tumoursphere and colony formation assay

For sphere-forming ability assays, INT-SFT or IEC139 cells were seeded in Corning^®^ Costar^®^ (NY, USA) Ultra-Low Attachment 96-Well Plate at 1.5 × 10^3^ cells/well. Then, images were obtained after 8 days using an inverted microscope Olympus IX-71. In 3D drug resistance assays, INT-SFT and IEC139 clones were plated at different densities, in order to obtain spheroids of comparable size at the time of treatment: 5 × 10^3^ cells/well for shNT and 10^4^ cells/well for shISG15. 4 days after plating, spheroids were properly formed and were then treated with either 20 µM pazopanib (Novartis, Basel, Switzerland), 0.5 nM trabectedin (PharmaMar, Madrid, Spain) or 50 nM doxorubicin (Sigma-Aldrich). Drug concentrations correspond approximately to 2X IC50 values for each drug according to cell viability in 2D cultures. After 72 h, drugs were removed. Images were acquired each day using an inverted microscope Olympus IX-71, until appreciable differences in regrowth: 12 days post-treatment for pazopanib and doxorubicin; 20 days post-treatment for trabectedin. Sphere size (area) was determined using the ImageJ tool *Analyze Spheroid Cell Invasion In 3D Matrix* (RRID:SCR_021204).

For colony formation assay 1 × 10^3^ INT-SFT cells were seeded in 100 mm plates. After 8 days, colonies were stained using methyl violet dye (Thermo Fischer Scientific) and counted.

#### Proliferation, migration, and invasion

A total of 10^3^ cells/well were seeded in 96-well plates and were left in the incubator to settle for 24 h. Each day (until day 6), 20 µl of CellTiter 96^®^ AQueous One Solution Cell Proliferation Assay (MTS) (Promega, Madison, WI, USA) was added to the media. After 30 min of incubation, absorbance at 490 nm was read using iMark microplate absorbance reader (Bio-Rad, Hercules, CA, USA). For wound healing assays, cells were cultured in 6-well plates until completely confluent. Then, a scratch was gently made across the well using a 200 µl pipette tip, cells and debris were rinsed using PBS. Subsequently, images were obtained with an Olympus IX-71 microscope at various time points. The area of the gap was quantified for each image using ImageJ software and the speed of migration was calculated at µm^2^/h. For invasion assays, cells were cultured in serum-free media for 24 h. Then, 10^4^ cells were seeded in the top chamber of 8 µm pore polycarbonate transwells (Corning Costar), previously treated overnight with 0.2X Cultrex Basement Membrane Extract (BME) in coating buffer (Trevigen, McKinley, MN, USA). Serum-containing media was used as chemoattractant in the bottom chamber. After 48 h, the top side of each polycarbonate insert was cleaned using a cotton swab and rinsed with PBS. The membrane was fixed for 5 min with 100% methanol at -20 ºC, then stained using DAPI reagent (Invitrogen) and mounted on a slide to be observed under fluorescent microscopy (Olympus BX-61). Cell nuclei were counted for each condition.

#### Apoptosis analysis

The number of apoptotic, early apoptotic, and necrotic cells was evaluated in INT-SFT/IEC139 sh non-targeting (shNT) and INT-SFT/IEC139 shISG15 cell lines, after 20 µM pazopanib, 0.5 nM trabectedin or 25 nM doxorubicin 72 h treatment. A FITC Annexin V Apoptosis Detection Kit with PI was used to determine cell death (Immunostep; Salamanca, Spain), following the manufacturer’s instructions. Apoptosis levels were determined by flow cytometry, FACSCanto™ II Cell Analyzer (BD Biosciences) and data analysed with both BD FACS Diva and FlowJo software.

#### RNA extraction and RT-qPCR

RNA was extracted from cell culture with the RNA PureLink RNA Mini Kit (Invitrogen, Carlsbad, CA, USA), quantified with the NanoDrop One C spectrophotometer (Thermo Fisher Scientific, Madison, WI, USA) and reverse transcribed to cDNA using the High-Capacity Reverse cDNA Transcription Kit (Applied Biosystems, Thermo Fischer Scientific, Foster City, CA, USA). Expression levels of the selected genes were measured by RT-qPCR, using the following TaqMan RNA probes (Applied Biosystems): *ISG15* (Hs00192713_m1), *SOX2* (Hs01053049_s1), *NANOG* (Hs04260366_g1), *ABCB1* (Hs01067802_m1), *MYC* (Hs00153408_m1) and *ALDH1A1* (Hs00946916_m1); *GAPDH* (Hs03929097_g1) was used as housekeeping gene for data normalisation. An ABI Prism 7900HT (Applied BioSystems) real time PCR system was used. The relative expression of genes was expressed using INT-SFT, IEC139 or shNT as a reference, depending on the experiment. ISG15 expression induction was performed in INT-SFT/IEC139 shNT or shISG15 cells after treatment with 250 U/ml human IFN-β1a (Miltenyi Biotec, Bergisch Gladbach, Germany), 20 µM pazopanib, 0.5 nM trabectedin for 48 h or 25 nM doxorubicin.

#### Western blot analysis

Cell lysis and protein extraction was carried out using the RIPA buffer [1 M Tris–HCl pH 8 (PanReac AppliChem, ITW Reagents), 0.5 M EDTA (Thermo Fisher Scientific), Triton™ X-100 (Sigma-Aldrich), 10% sodium deoxycholate (Sigma-Aldrich), 10% SDS (Sigma-Aldrich) and 3 M NaCl (Thermo Fisher Scientific)], supplemented with protease and phosphatase inhibition cocktails (Sigma-Aldrich). 20 µg protein sample were separated by SDS-PAGE, then transferred to 0.2 µm pore-size nitrocellulose membranes (Bio-Rad). Membranes were blocked for 1 h using 5% BSA (PanReac AppliChem, ITW Reagents), in 1X TBS-T (0.1% Tween20, Bio-Rad). Subsequently, the following antibodies were incubated in the same buffer for 16 h at 4 ºC: ISG15 (Santa Cruz Biotechnology, sc-166755), α-tubulin (Sigma-Aldrich, T9026). Membranes were washed with 1X TBS-T, then incubated with Rabbit Anti-Mouse IgG–Peroxidase antibody (Sigma-Aldrich) or Goat Anti-Rabbit IgG H&L peroxidase-conjugated antibody (Abcam, Cambridge, UK). HRP substrate was used for chemiluminescent detection and image acquisition was performed using a Chemidoc Imaging System (Bio-Rad). ISG15 expression was relativised against INT-SFT values.

### Immunofluorescence (IF)

A total of 2 × 10^4^ cells were plated on round glass coverslips contained in 24-well plates. After 24 h incubation, coverslips were fixed with 3% paraformaldehyde (PFA) and then permeabilised using 0.2% Triton X-100 (Sigma-Aldrich) 2X PBS. For ER staining, cells were exposed in vivo for 30 min with 1X ER Staining Kit—Cytopainter (Abcam), prior to fixation and permeabilization. Blocking was done with 1% BSA 2X PBS for 30 min RT. ISG15 (Santa-Cruz) 1:200 was incubated at 4 ºC overnight. The following secondary fluorescent antibodies were used at 1:1000 for 1 h at RT: Goat anti-Rabbit IgG (H + L) Cross-Adsorbed Secondary Antibody, Alexa Fluor 488 and Goat anti-Mouse IgG1 Cross-Adsorbed Secondary Antibody, Alexa Fluor 546 (Invitrogen). Nucleic acid stain DAPI (Invitrogen) was subsequently added for 15 min. After careful washing, coverslips were mounted onto microscope slides with ProLong™ Gold mounting media (ThermoFisher). Fluorescence microscope Olympus BX-61 was used for image acquiring. For ER/ISG15 colocalization Z-stack images were obtained using microscope Leica SP5 (Leica, Wetzlar, Germany). For spheroid IF, 2 µM Calcein AM (Cayman Chemical, Ann Arbor, MI, USA) and 33 µM Hoechst 33,342 (Abcam) were incubated for 3 h in cell culture incubator. Subsequently, spheroids were visualized at confocal microscope Leica SP5, using the same acquisition parameters for independent experiments. Dyes were not required to be washed away [32, 33]. For signal quantification, intensity density (IntDen)/spheroid area ratio was determined using ImageJ.

## Results

### ISG15 expression is associated with worse prognosis in M-SFT patients

ISG15 was analysed in samples of 49 SFT patients. The median age at diagnosis was 63, with female predominance (59% vs 41%). The original study distinguished subtypes based on histologic criteria, i.e., malignant/dedifferentiated (51% and 4% respectively) and typical (45%) SFT. Based on the 3-variable risk model classification criteria, intermediate risk was predominant (53%) followed by low risk (33%) and high risk (12%), one patient was non-evaluable (2%) Patients harboured NAB2ex6-STAT6ex16/17 (47%), NAB2ex4-STAT6ex2 (31%) or other (22%) gene fusion variants.

**Fig. 1:**
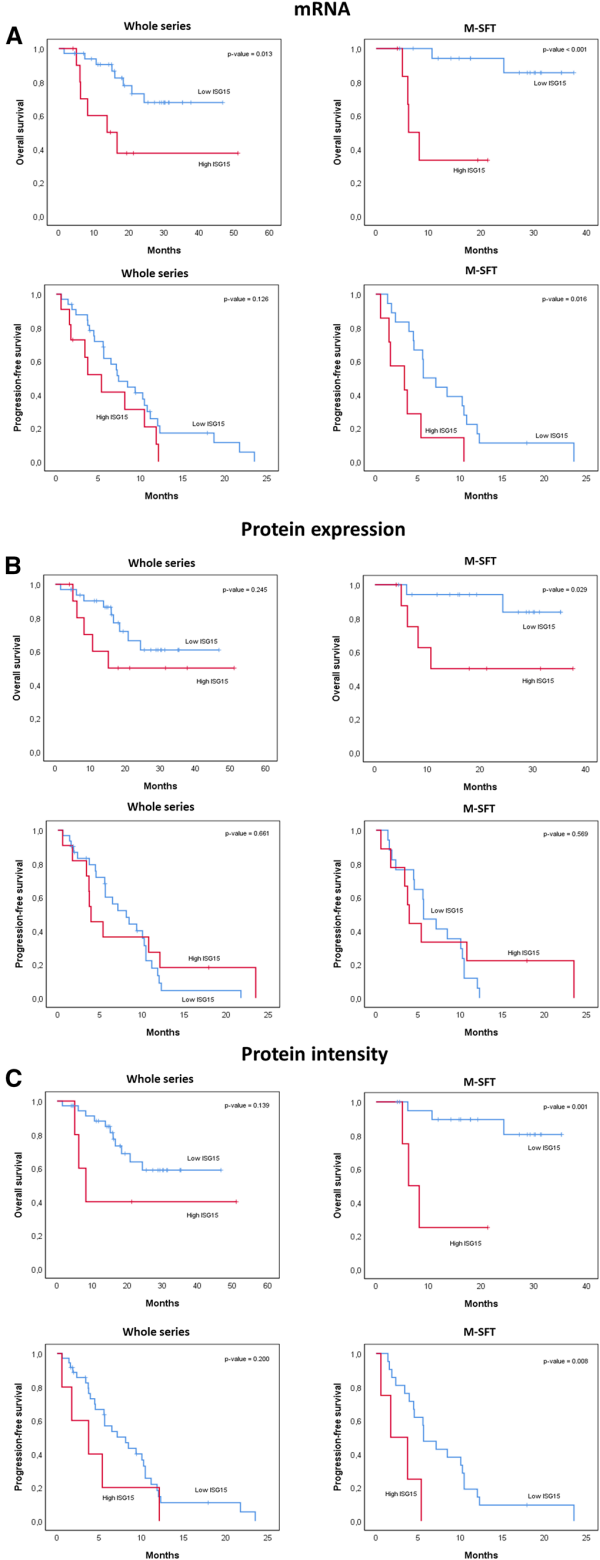
Solitary fibrous tumour survival analysis by ISG15 levels. Tables show data for patients enrolled in the GEIS32 study. **A** Overall survival (OS) and progression-free survival (PFS) according to Choi criteria by *ISG15* gene expression. Upper quartile Q3 was considered the cut-off value to discriminate between High and Low *ISG15* expression groups. **B** OS and PFS according to Choi criteria by ISG15 protein extension. Patients were considered as Low ISG15 when ISG15 extension by IHC was < 25% and High ISG15 when protein extension was > 25%. **C** OS and PFS according to Choi criteria by ISG15 protein intensity. Samples with negative or weak staining were considered Low ISG15 and strong staining were included in the High ISG15 group. Significance between groups was defined at *p*-values < 0.05

In the univariate analysis, higher ISG15 gene expression significantly correlated with worse OS [13.8 months (95% CI 2.2–25.3) vs NA], but not with PFS. When focusing on the M-SFT cohort, high ISG15 mRNA levels correlated with worse OS (NA) and worse PFS [3.4 months (95% CI 0.0–7.7) vs 5.6 months (95% CI 3.5–7.8)] (Fig. 1A). Moreover, *ISG15* gene expression also positively correlated with number of mitoses (Pearson = 0.663, *p*-value < 0.001). Immunohistochemical analyses indicated that the percentage of positive stained cells for ISG15 protein was negative in 40% of patients (17/43), + in 35% (15/43), +  + in 19% (8/43) and +  +  + in 7% (3/43). ISG15 protein intensity was negative in 40% (17/43), weak in 49% (21/43) and strong in 12% (5/43) (Table 1). ISG15 localisation was observed at nuclear and/or cytoplasmic level (Supplementary Fig. 1F).

**Table 1:** Baseline characteristics (*n* = 49)

Median age (Range)	63 (31, 87)
Gender (M/F)	20 (41%)/29 (59%)
ECOG PS at baseline	
0	28 (57%)
1–2	21 (43%)
Extension at diagnosis	
Localized	30 (61%)
Locally advanced	3 (6%)
Metastatic	16 (33%)
Extension at baseline^a^	
Locally advanced	7 (14%)
Metastatic	42 (86%)
Median months to M1 (Range)	34 (0, 302)
Median tumour size at baseline (Range, cm)	76 (11, 415)
Number of mitoses/10 HPF	
0–3	25 (51%)
> 3	24 (49%)
Original cohorts	
Typical	22 (45%)
Malignant	25 (51%)
Dedifferentiated	2 (4%)
Risk model (3 variable)	
Low risk	16 (33%)
Intermediate risk	26 (53%)
High risk	6 (12%)
Non-evaluable	1 (2%)
*NAB2/STAT6* breakpoint fusion	
ex4-ex2	14 (31%)
ex6-ex16/17	21 (47%)
Other	10 (22%)
ISG15 protein expression (JM)	
Negative	17 (35%)
5–25%	15 (31%)
25–50%	8 (16%)
> 50%	3 (6%)
Non-evaluable	6 (12%)
ISG15 protein intensity (JM)	
Negative	17 (35%)
Weak	21 (43%)
Strong	5 (10%)
Non-evaluable	6 (12%)

*JM* Jose Merino-Garcia evaluation

^a^Before enrolment in GEIS-32 trial (ClinicalTrials.gov, NCT02066285)

High ISG15 protein expression correlated with worse OS in M-SFT patients (NA; Fig. 1B), even though the prognostic value of ISG15 protein expression was not validated for the whole series. Similarly, in the M-SFT cohort ISG15 protein intensity correlated with worse OS [6.2 months (95% CI, 3.0–9.3) vs NA] and worse PFS [1.7 months (95% CI, 0.0–4.8) vs 5.6 months (95% CI, 3.3–8.0); Fig. 1C, Table 2 and 3]. Furthermore, high and low ISG15 groups for gene expression and staining intensity showed a positive correlation by *χ*^2^ analysis. ISG15 did not show any prognostic value for the T-SFT cohort (Supplementary Fig. 1).

**Table 2:** Univariate analyses (Log-rank) of clinicopathological factors according to progression-free survival and overall survival in the whole series

	Median PFS (CHOI) (95% CI)	*p*	Median PFS (Local) (95% CI)	*p*	Median OS (95% CI)	*p*
Age						
0–63	10.1 (7.0, 13.2)	0.760	10.1 (5.6, 14.5)	0.321	NA	0.522
> 63	5.6 (4.0, 7.2)	–	5.4 (3.6, 7.2)	–	49.8 (13.8, 85.7)	–
Sex						
Male	7.1 (4.8, 9.5)	0.313	7.4 (3.1, 11.8)	0.267	24.3 (NA, NA)	0.237
Female	10.1 (5.7, 14.5)	–	10.1 (2.9, 17.2)	–	49.8 (NA, NA)	–
Size at baseline						
0–76	10.3 (5.0, 15.6)	0.247	11.2 (5.2, 17.1)	0.290	NA	0.006
> 76	7.4 (3.5, 11.3)	–	6.2 (3.8, 8.7)	–		–
Metastasis-free Interval					20.8 (12.1, 29.5)	
0–34	5.6 (2.0, 9.2)	0.114	5.6 (2.4, 8.8)	0.226	NA	0.079
> 34	10.5 (9.0, 12.0)	–	11.2 (9.6, 12.7)	–	49.8 (13.8, 85.7)	–
ECOG						
0	10.5 (5.7, 15.2)	0.121	11.9 (9.8, 13.9)	0.041	NA	0.005
1–2	6.5 (2.4, 10.6)	–	5.6 (3.0, 8.3)	–	18.4 (10.0, 26.7)	–
Mitoses						
0–3	10.1 (6.3, 13.8)	0.010	10.1 (5.3, 14.9)	0.135	49.8 (7.9, 91.6)	0.565
> 3	5.6 (4.2, 6.9)	–	5.6 (3.5, 7.7)	–	NA	–
Extension at baseline						
Locally advanced	7.4 (4.6, 10.2)	0.802	9.6 (5.9, 13.4)	0.779	20.8	0.928
Metastatic	7.2 (4.4, 10.1)		7.2 (4.0, 10.4)	–	49.8	–
NAB2/STAT6 breakpoint fusion						
ex4-ex2	5.6 (2.5, 8.6)	0.183	4.0 (1.4, 6.6)	0.484	NA	0.691
ex6-ex16/17	10.5 (7.7, 13.3)	–	11.2 (8.3, 14.2)	–	NA	–
Other	10.1 (6.3, 13.9)	–	10.1 (6.4, 13.8)	–	49.8 (NA, NA)	–
*ISG15* Gene expression						
Below Q3	7.4 (4.8, 10.0)	0.126	9.6 (4.5, 14.8)	0.043	NA	0.013
Above Q3	5.4 (2.5, 8.3)	–	3.7 (0.1, 7.4)	–	13.8 (2.2, 25.3)	–
ISG15 Protein expression						
0–25%	9.6 (5.6, 13.7)	0.661	9.6 (5.6, 13.7)	0.954	NA	0.245
> 25%	5.4 (3.4, 7.4)	–		–	15.1 (2.0, 28.3)	–
ISG15 Protein intensity			5.4 (3.4, 7.4)			
Negative/low	8.1 (4.4, 11.9)	0.200	8.8 (2.9, 14.6)	0.087	NA	0.139
Strong	3.7 (0.0, 8.0)	–	3.7 (0.0, 8.0)	–	8.2 (3.8, 12.6)	–

**Table 3:** Univariate analyses (Log-rank) of clinicopathological factors according to progression-free survival and overall survival in M-SFT cohort

	HR PFS (CHOI) (95% CI)	*p*	HR PFS (Local) (95% CI)	*p*	HR OS (95% CI)	*p*
Age						
0–63	10.1 (7.0, 13.1)	0.184	10.6 (3.7, 17.4)	0.102	NA	0.539
> 63	4.5 (3.4, 5.6)	–	4.5 (3.5, 5.4)	–	NA	–
Sex						
Male	5.6 (5.5, 5.7)	0.826	5.6 (0.0, 12.9)	0.801	NA	0.673
Female	4.5 (2.8, 6.3)	–	5.4 (3.0, 7.7)	–	NA	–
Size at baseline						
0–76	5.6 (2.6, 8.7)	0.140	5.6 (0.7, 10.5)	0.195	NA	0.361
> 76	5.4 (3.4, 7.3)	–	5.4 (2.7, 8.0)	–	NA	–
Metastasis-free Interval						
0–34	4.5 (2.2, 6.8)	0.728	4.5 (2.1, 6.9)	0.732	NA	0.191
> 34	8.5 (3.4, 13.5)	–	10.1 (2.7, 17.4)	–	NA	–
ECOG						
0	7.1 (1.2, 13.1)	0.042	10.6 (4.7, 16.5)	0.012	NA	0.154
1–2	4.5 (2.1, 6.8)	–	4.5 (2.1, 6.8)	–	NA	–
Mitoses						
0–3 (only 3 cases)	10.1 (1.2, 18.9)	0.266	10.1 (1.2, 18.9)	0.302	NA	0.319
> 3	5.6 (4.2, 6.9)	–	5.6 (3.5, 7.7)	–	NA	–
Extension at baseline						
Locally advanced						
Metastatic	All metastatic	All metastatic	All metastatic	All metastatic	All metastatic	
NAB2/STAT6 breakpoint fusion						
ex4-ex2	3.9 (2.4, 5.5)	0.526	4.0 (2.2, 5.8)	0.663	NA	0.926
ex6-ex16/17	8.5 (3.5, 13.5)	–	10.6 (3.1, 18.0)	–	NA	–
Other	7.1 (1.4, 12.9)	–	8.8 (0.0, 18.0)	–	NA	
*ISG15* Gene expression						
Above Q3	5.6 (3.5, 7.8)	0.016	8.8 (2.2, 15.4)	0.068	NA	< 0.001
Above Q3	3.4 (0.0, 7.7)	–	3.4 (0.0, 7.7)	–	NA	
ISG15 Protein expression						
0–25%	5.6 (3.5, 7.7)	0.569	8.8 (0.37, 17.2)	0.885	NA	0.029
> 25%	3.9 (3.3, 4.5)	–	4.0 (3.2, 4.8)	–	NA	–
ISG15 Protein intensity						
Negative/low	5.6 (3.3, 8.0)	0.008	8.8 (2.6, 15.0)	0.008	NA	0.01
Strong	1.7 (0.0, 4.8)	–	1.7 (0.0, 4.8)	–	6.2 (3.0, 9.3)	–

### ISG15 is overexpressed in M-SFT cell line versus other STS cell lines

**Fig. 2:**
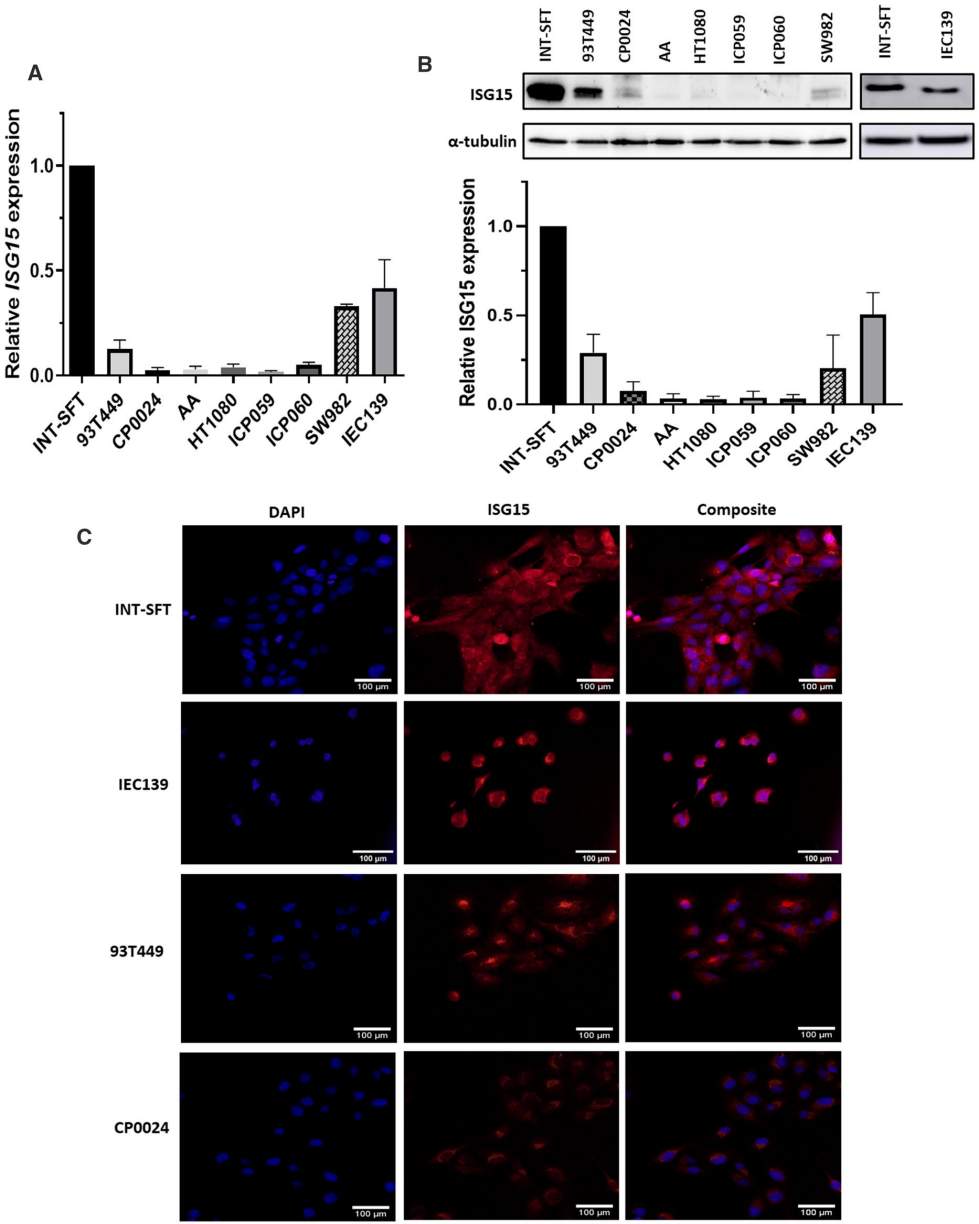
ISG15 expression in sarcoma cell lines.** A** ISG15 RT-qPCR on RNA extracts of different sarcoma subtype cell lines. Extracts were collected at equal confluence, 48 h after cells were seeded. Solitary fibrous tumour INT-SFT cell line greatly expresses ISG15, followed by IEC139 primary SFT line, when compared to other subtypes (*n* = 4). **B** ISG15 immunoblot on analogous protein extracts. ISG15 gene expression seems to correspond to its mRNA levels (*n* = 4). **C** IF microscopy images showing nuclei (DAPI) in blue, ISG15 in red and overlapping images. Pictures were taken with exact same exposure time. ISG15 shows at both cytoplasmic and nuclear compartments, with accumulations at areas adjacent to nuclei (*n* = 3)

ISG15 expression was determined, both at mRNA and protein levels, in a panel of sarcoma cell lines: INT-SFT, IEC139, 93T449, CP0024, AA, HT1080, ICP059, ICP060 and SW982. *ISG15* gene expression levels were significantly higher, in INT-SFT cell line followed by IEC139 (both M-SFT) against other sarcoma subtypes (Fig. 2A). Specifically, INT-SFT presented 3.0- and 7.9-fold higher ISG15 mRNA levels, as compared to SW982 and 93T449 cell lines respectively. When compared to CP0024, AA, HT1080, ICP059 and ICP060, INT-SFT showed 19.6- to 55.6-fold higher *ISG15* gene expression. IEC139 expressed between 8.2- and 23.7-fold more ISG15 mRNA levels than these lines and 3.3-fold more than 93T449. Analogous results were obtained at protein level (Fig. 2B). Similarly, INT-SFT showed 4.9- to 33.3-fold more ISG15 protein, when compared to other sarcoma subtypes, but only 1.98-fold more when compared to IEC139. ISG15 expression is seemingly not related to SV40 LargeT antigen transformation, as no ISG15 is observed in HEK293T cells (Supplementary Fig. 1). Of note, ISG15 appeared to be differentially overexpressed in two sarcoma datasets (Tumor Sarcoma Mesenchymal—Boshoff—96 and Tumor Sarcoma—Filion—137) versus a normal tissue repository (Normal Various (GNF)—Su—158) (Supplementary Fig. 1), supporting potential interest of ISG15 expression in sarcoma. In SFT, ISG15 expression is mainly cytoplasmic, with accumulation at vesicles and areas adjacent to nuclei, which does not colocalize with endoplasmic reticulum (Supplementary Fig. 1G). However, nuclear location can also be observed (Fig. 2C).

### ISG15 enhances CSC phenotype in M-SFT cell line

**Fig. 3:**
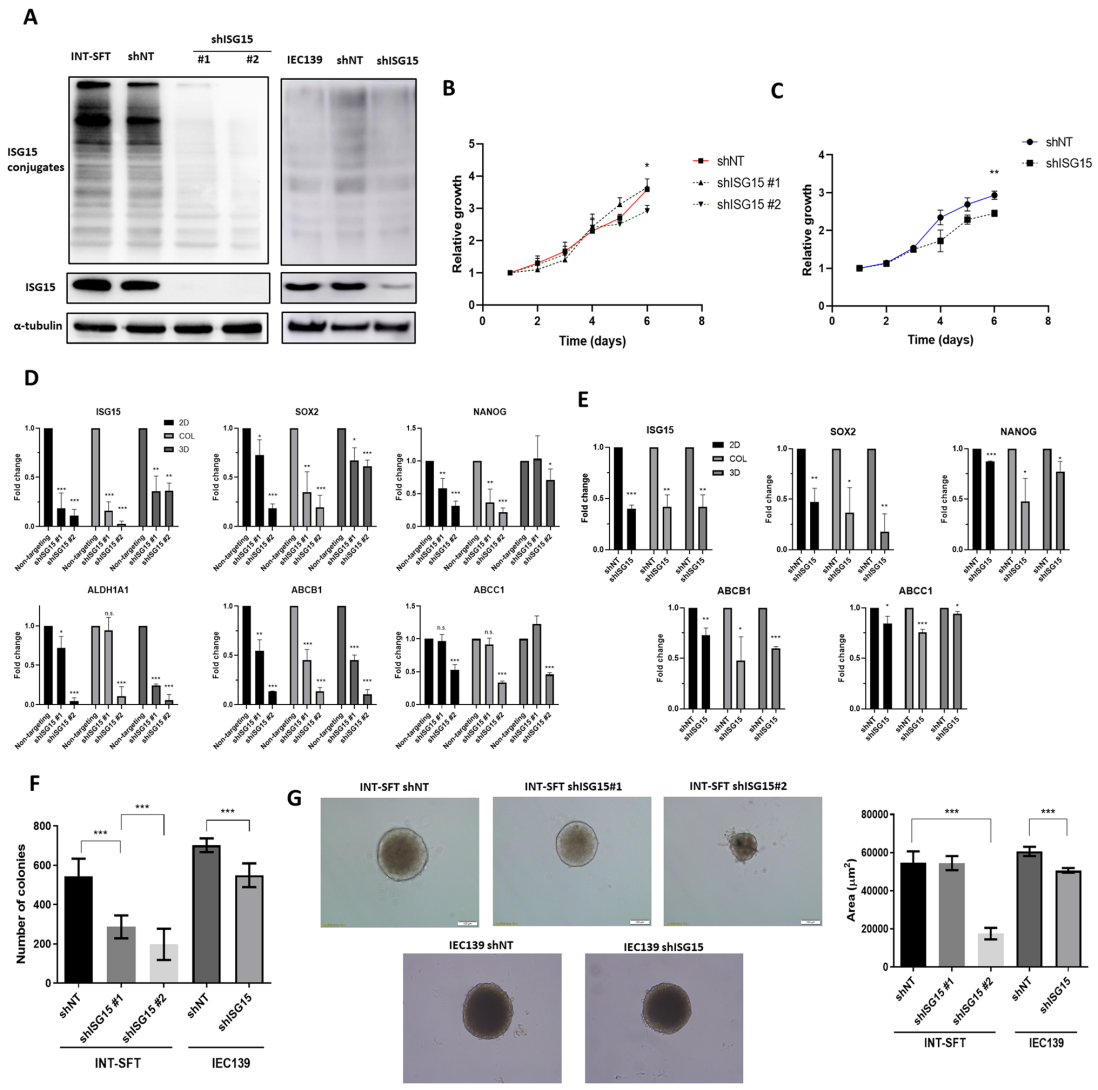
ISG15 downregulation in SFT cells inhibits sarcoma CSC markers expression and CSC properties.** A** ISG15 knockdown by shRNA-containing transduction particles. shNT contains a non-target shRNA sequence and corresponds to knockdown control. By protein immunoblot no visible ISG15 bands are observed for either INT-SFT shISG15#1 or shISG15#2. IEC139 shISG15 presents a band of around 60% less intensity. ISG15 conjugates are significantly diminished for INT-SFT but not IEC139 cells (*n* = 3) **B** Proliferation assay showing statistically significant differences at day 6 between INT-SFT shNT and shISG15#2 and **C** IEC139 shNT vs shISG15 cell lines. Number of cells was induced by reading absorbance at 490 nm after 20 min MTS reagent exposure. Proliferation was represented relative to day 1 signal (*n* = 4). **D** RT-qPCR from 2D, colony culture and 3D-spheroid extracts; the latter two are enriched in CSC cells. INT-SFT shISG15#2 shows a greater ISG15 knockdown by gene expression. *SOX2, NANOG, ALDH1A1, ABCB1* and *ABCC1* sarcoma CSC markers are downregulated in silenced cells (*n* = 3). **E** CSC-related gene expression is also inhibited in IEC139 shISG15 cells. **F** Colony formation ability is impaired by ISG15 silencing. 1000 cells/condition were seeded in 10 mm plates then, after 8 days in the incubator, colonies were stained with methyl violet and counted (*n* = 4). **G** Spheroid forming ability is reduced for INT-SFT shISG15#2, which corresponds to greater ISG15 knockdown. IEC139 shISG15 spheroids are also of smaller size. 1500 cells/condition were seeded, after 8 days images were obtained, and sphere size was quantified (*n* = 3). *T*-student tests: (*) stands for *p*-value < 0.05, (**) for *p*-value < 0.01 and (***) for *p*-value < 0.001. Error bars are indicative of means ± SD. *n.s.* not significant

INT-SFT and IEC139 cells were transduced with lentiviral particles containing shRNA against ISG15 (shISG15) and non-targeting shRNA (shNT) for control. Two independent shISG15 clonogenic cell lines, shISG15#1 and shISG15#2, were established for INT-SFT. In the case of IEC139, a shNT and a shISG15 clonogenic cell lines were established. In 2D cultures ISG15 mRNA levels for INT-SFT shISG15#1 and shISG15#2 decreased by 81.5% and 88.7%, respectively, when compared to control shNT cells (Fig. 3B). The free form of ISG15 appeared to be almost completely depleted, as no bands could be observed by WB. In addition, ISG15 conjugates were reduced by 66.3% and 75.5%, for shISG15#1 and shISG15#2 respectively (Fig. 3A). Also, the amount of ISG15 secreted to the medium was markedly decreased (Supplementary Fig. 2). ISG15 silencing for IEC139 was not as optimal, with a 60.2% ISG15 decrease at mRNA level and a 56.7% at protein level. No significant differences were observed for ISG15 conjugates. In our experimental conditions, downregulation of ISG15 inhibited cell proliferation. Significant differences in cell growth were observed at day 6 after plating for shISG15#2 vs shNT INT-SFT cells and for shISG15 vs shNT IEC139 cells (Fig. 3B, C). However, ISG15 knockdown did not seem to affect cell migration or invasion (Supplementary Fig. 2C, D). Besides, CSC and drug-resistance related genes in sarcoma such as *SOX2, NANOG*, *ALDH1A1, ABCB1* and *ABCC1* were down-regulated in 2D, as well as in CSC-enriched cultures (colony-forming and 3D/ spheroid). To be precise, reduction percentages in 2D cultures for INT-SFT shISG15#2 cells were of 81.7% for *SOX2*, 68.9% for *NANOG*, 95.4% for *ALDH1A1,* 86.3% for *ABCB1*, and 47% for *ABCC1*, in reference to shNT cells*.* Likewise, in colony-forming cultures of shISG15#2 cells, gene expression was decreased by 80.6% for *SOX2*, 78.3% for *NANOG*, 89.1% for *ALDH1A1*, 86.6% for *ABCB1*, and 66.3% for *ABCC1.* Lastly, in shISG15#2 3D-spheroid gene-expression reduction was of 38.8% for *SOX2*, 29.1% for *NANOG*, 94.2% for *ALDH1A1*, 89.5% for *ABCB1*, and 54.3% for *ABCC1,* compared to shNT (Fig. 3D). Besides, *MYC* was only significantly downregulated in the 3D group (32.2%; Supplementary Fig. 2E). For IEC139 we observed similar results. In 2D cultures expression was reduced a 52.8% for *SOX2,* 12.8% for *NANOG,* 27.1% for *ABCB1* and 15.8% for *ABCC1*; in colony-forming cultures 63.2% for *SOX2*, 52.4% for *NANOG,* 52.3% for *ABCB1* and 24.5% for ABCC1; and in 3D cultures 82.2% for *SOX2,* 22.7% for *NANOG*, 40.0% for *ABCB1* and 5.8% for *ABCC1.* No *ALDH1A1* expression was detected for IEC139 in our experimental conditions. Furthermore, colony formation and 3D tumour spheroid assembly, which are both considered CSC characteristics in vitro*,* were accordingly impaired in shISG15 for both M-SFT cell lines (Fig. 3F, G; Supplementary Fig. 2B). Namely, INT-SFT shISG15#1 and shISG15#2 cells formed less colonies (286.9 ± 58.3 & 197.6 ± 79.5) than control (544.0 ± 89.8) in clonogenic cultures. Similarly, IEC139 shISG15 cells presented less colony-forming ability (549.3 ± 60.2) than shNT (702.0 ± 35.1). In addition, tumour spheroids grown in round-bottom low-attachment plates were smaller in size for INT-SFT shISG15#2 (17,511 µm^2^ ± 2985) versus INT-SFT shNT cells (54,612 µm^2^ ± 6005) and for IEC139 shISG15 (50,639 µm^2^ ± 1236) versus IEC139 shNT (60,600 µm^2^ ± 2414). As ISG15 knockdown in INT-SFT shISG15#2 compared to shISG15#1 exhibited lower levels of *ISG15* mRNA and conjugates, together with a greater inhibition of CSC phenotype (showed by lower stem-marker levels and decreased capacity to form colonies and spheroids), further assays were performed using only this clone (Fig. 3D, F, G).

**Fig. 4:**
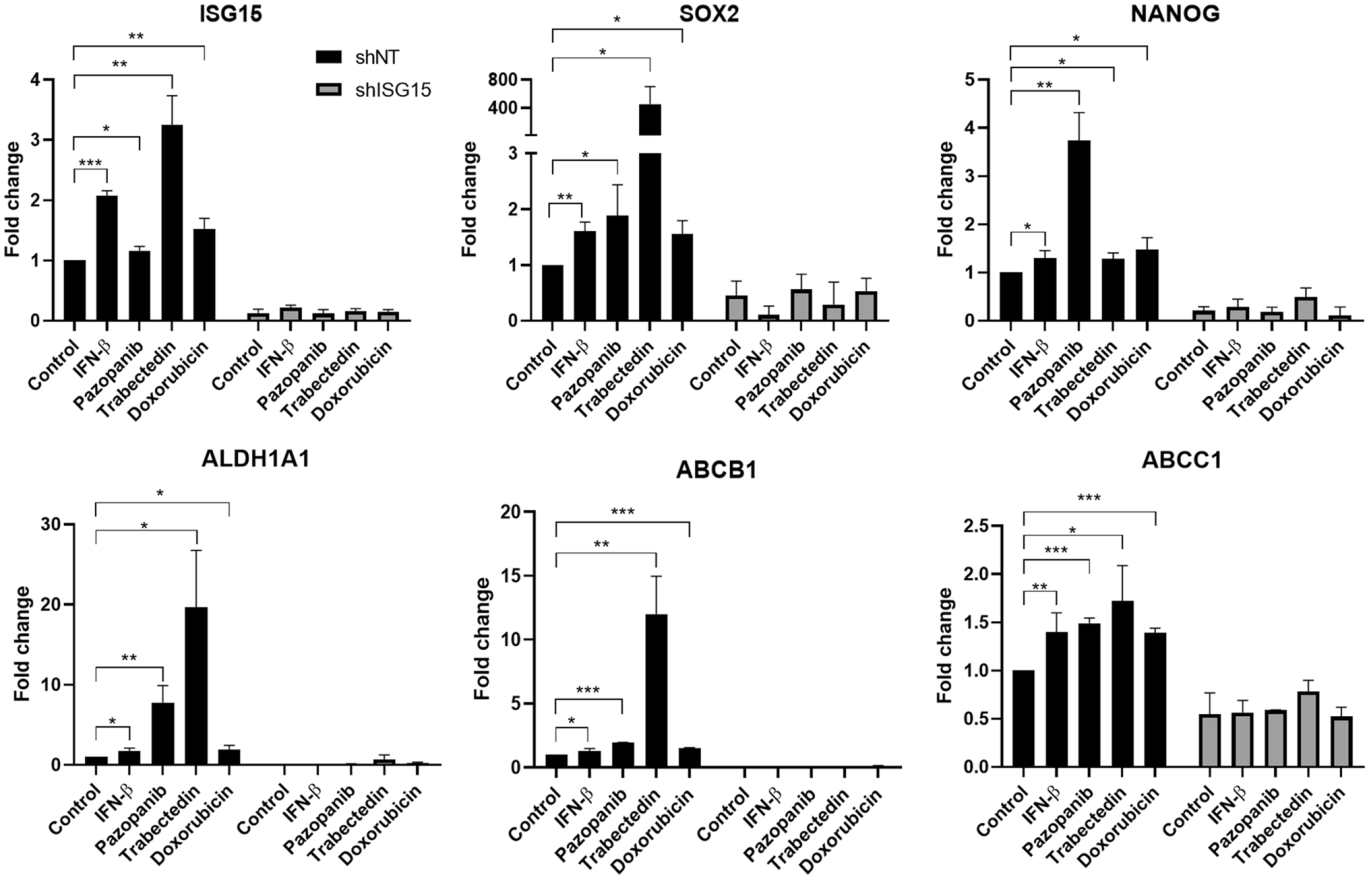
ISG15 is upregulated by IFN-β or drug treatment and enhances CSC markers’ gene expression. ISG15 induction for 48 h by either 250 U/mL IFN-β or drug treatment (20 µM pazopanib, 0.5 nM trabectedin or 25 nM doxorubicin) promotes gene expression of *SOX2, NANOG, ALDH1A1, ABCB1* and *ABCC1* genes related with CSC behavior in sarcoma, in INT-SFT shNT control cells. This is not observed for shISG15 knockdown cells. *T*-student tests: (*) stands for *p*-value < 0.05, (**) for *p*-value < 0.01 and (***) for *p*-value < 0.001. Error bars are indicative of means ± SD. *N.s.* not significant

In control INT-SFT cells (shNT), treatment with either IFN-β (as a well-known ISG15 inductor), pazopanib, trabectedin or doxorubicin results in an overexpression of several of the stem markers described above: *SOX2, NANOG, ALDH1A1*, *ABCB1* and *ABCC1*. Cells exposed to trabectedin show a sharper increase in the expression of most of these genes (*SOX2* = 451.0 ± 250.8, *NANOG* = 1.283 ± 0.125, *ALDH1A1* = 19.67 ± 7.088, *ABCB1* = 12.01 ± 2.952, *ABCC1* = 1.723 ± 0.365) compared to IFN-β (*SOX2* = 1.607 ± 0.161, *NANOG* = 1.292 ± 0.163, *ALDH1A1* = 1.743 ± 0.358, *ABCB1* = 1.286 ± 0.186, *ABCC1* = 1.397 ± 0.201), pazopanib (*SOX2* = 1.883 ± 0.557, *NANOG* = 3.740 ± 0.579, *ALDH1A1* = 7.775 ± 2.137, *ABCB1* = 1.947 ± 0.005, *ABCC1* = 1.491 ± 0.054) or doxorubicin (*SOX2* = 1.550 ± 0.246, *NANOG* = 1.474 ± 0.247, *ALDH1A1* = 1.912 ± 0.560, *ABCB1* = 1.493 ± 0.051, *ABCC1* = 1.394 ± 0.047, Mean fold-change values for treated group vs control). This also corresponds with a higher ISG15 induction for trabectedin (3.254 ± 0.482) compared to IFN-β (2.073 ± 0.086), pazopanib (1.161 ± 0.075) or doxorubicin treatment (1.529 ± 0.168, Mean fold-change values for treated group vs control). In contrast, ISG15 knock-down by shRNA prevents (i.e. *ABCB1, SOX2*) or greatly reduces (i.e. *ALDH1A1*) the induction of stem markers by either treatment in INT-SFT shISG15 cells (Fig. 4).

For IEC139 cells, ISG15 silencing was not substantial enough to prevent induction of the protein by pazopanib, trabectedin or doxorubicin exposure. This translates to sarcoma CSC-related markers (SOX2 being the most reliable) to be upregulated in both shNT and shISG15 when cells were treated with each drug (Supplementary Fig. 2F).

In addition, transcriptomic data on ISG15 silencing was obtained using the microarray Clariom S Assay, human. A gene set enrichment analysis (GSEA) was performed comparing INT-SFT shISG15 and INT-SFT shNT cells. Downregulation of various genes belonging to key CSC pathways was observed, like epithelial-mesenchymal transition, TGF-β, p53 and myogenesis. Interestingly, a significant increase was shown in genes related with KRas, MYC or MTORC1 (Supplementary Fig. 2G).

### Drug resistance is related to ISG15 expression

**Fig. 5:**
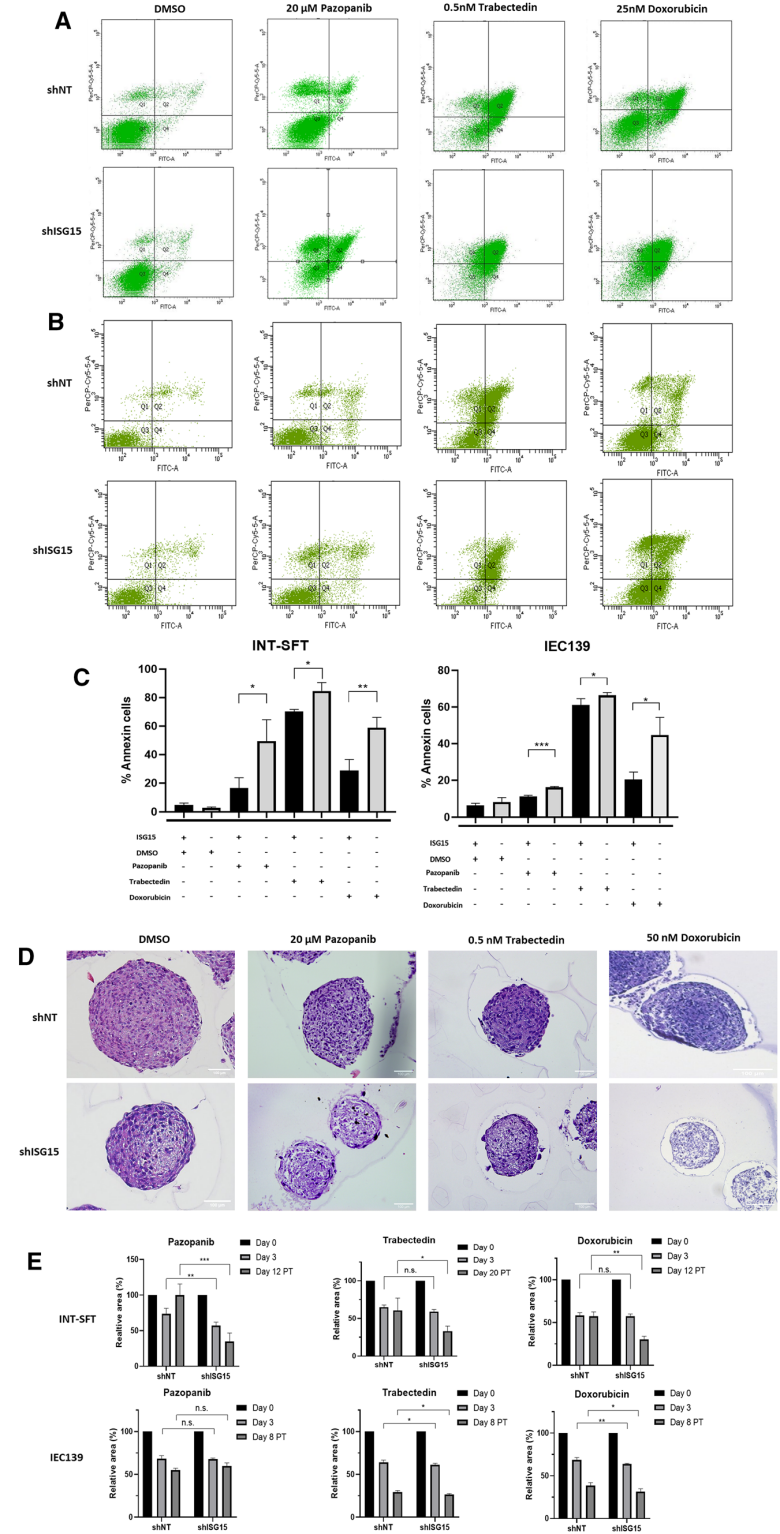
ISG15-downregulated solitary fibrous tumour 2D and 3D cultures are more sensitive to drug treatment. AnnexinV-FITC/IP apoptosis assay showing increased cell death for **A** INT-SFT or **B** IEC139 shISG15 vs shNT after 72 h with pazopanib, trabectedin or doxorubicin treatments (*n* = 3). **C** Annexin V-positive percentage of cells which correspond to apoptotic cells for each condition. **D** shISG15 haematoxylin/eosin-stained spheroid samples show increased necrotic area after 72 h drug exposure when compared to shNT spheres (*n* = 2). **E** Spheroids were treated with each drug (day 0), then were released from each drug after 72 h treatment (day 3), images of live spheroids were obtained at day 12 or 20 post-treatment for pazopanib and doxorubicin or trabectedin respectively. For IEC139 cells, images were obtained until day 8 post-treatment. Relative area to day 0 for each individual spheroid is represented (*n* = 4)**.** *T*-student tests: (*) stands for *p*-value < 0.05, (**) for *p*-value < 0.01, (***) for *p*-value < 0.001

Apoptosis was augmented in shISG15 vs shNT, when M-SFT cells were treated with 20 µM pazopanib (recommended first line antiangiogenic in SFT), 25 nM doxorubicin (first line chemotherapy in STS) or 0.5 nM trabectedin (second line treatment in STS) for 72 h (Fig. 5A, C; Supplementary Fig. 3). To be precise, apoptotic (Annexin V-positive) cell populations showed significant increases of 2.99-fold for pazopanib, 1.20-fold for trabectedin and 2.04-fold for doxorubicin treatments, in INT-SFT shISG15 compared to INT-SFT shNT cells (Fig. 5A, C). Likewise, apoptosis was significantly augmented in IEC139 shISG15 cells by 1.42-fold for pazopanib, 1.10-fold for trabectedin and 2.19-fold for doxorubicin treatments (Fig. 5B, C). In parallel, drug resistance was tested in 3D spheroid cultures. At 72 h with pazopanib, INT-SFT shISG15 spheroids exhibited a more drastic shrinkage in area (43.1%) than INT-SFT shNT spheroids (26.3%), due to increased cell death (Fig. 5E, Supplementary Fig. 4). However, no significant size differences were observed, between silenced and control cells, at 72 h for trabectedin or doxorubicin treatments. Besides, haematoxylin–eosin staining revealed a more pronounced fibrotic/necrotic state of shISG15 spheres after pazopanib, trabectedin and doxorubicin treatment (Fig. 5D). In contrast, IEC139 shISG15 spheroids did show a significant reduction in area compared to control, after 72 h trabectedin or doxorubicin, but not for pazopanib treatment (Fig. 5D).

**Fig. 6:**
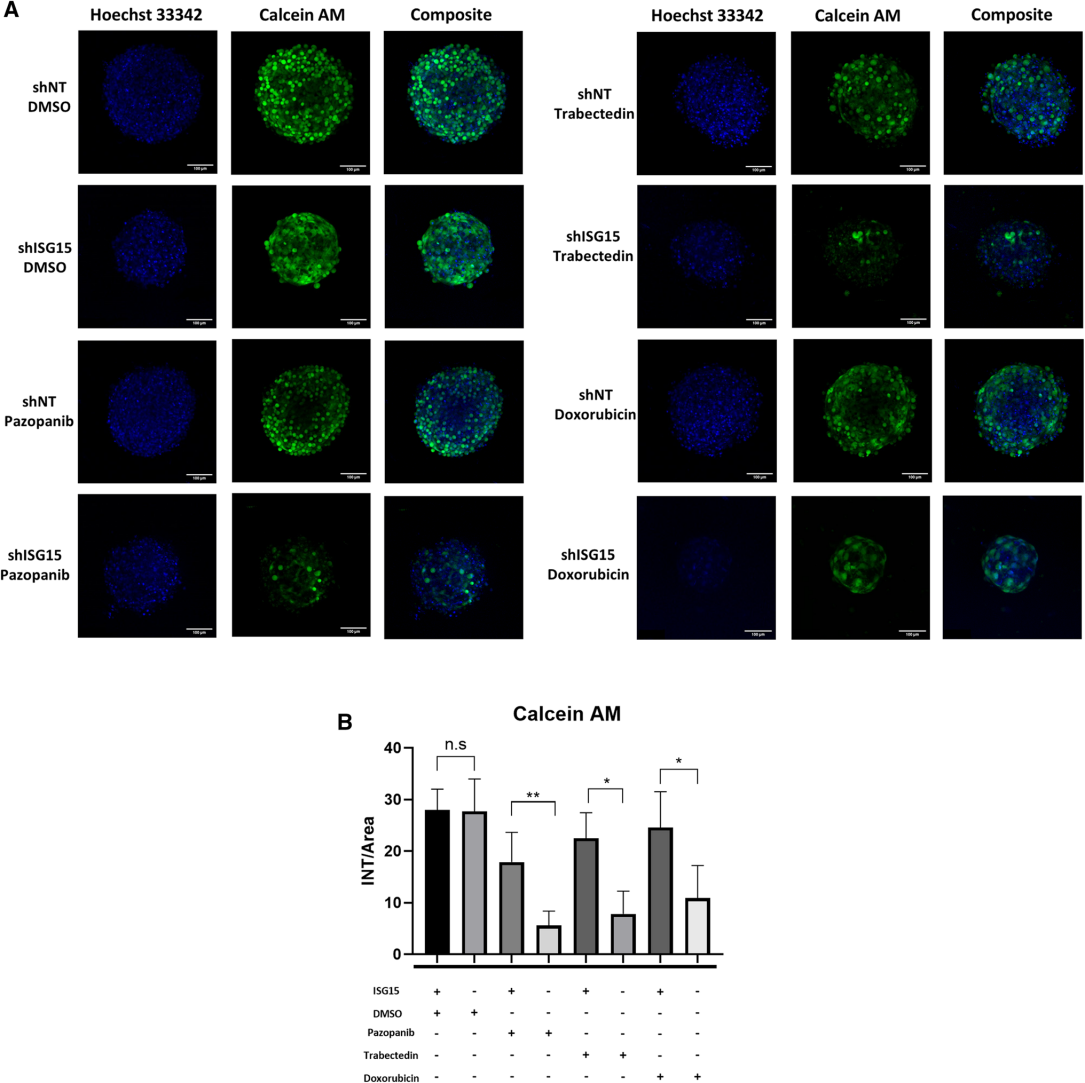
ISG15 knockdown tumour-spheres are more sensible to drug treatment. **A** Hoecsht 33,342 and Calcein AM staining, marking nuclei and live cells respectively, was performed on control (DMSO) and treated tumour spheroids for 3 h at 37 ºC. Images correspond to maximum projection of Z-stack, acquired using confocal microscopy. Independent experiments were performed (*n* = 5). **B** Quantification using ImageJ of Calcein AM intensity/area for each condition

In addition, INT-SFT 3D spheroids were stained using Hoechst 33,342 and Calcein AM which mark nuclei and live cells respectively. Concordantly, the raw intensity signal of Calcein AM/area ratio was significantly lower in ISG15 deprived spheroids versus control, after 72 h drug treatment (Fig. 6). Specifically, Calcein AM signal/area ratio was 68.8% lower in pazopanib, 65.1% in trabectedin and 55.6% in doxorubicin treatments, for shISG15 compared to shNT spheroids. No significant differences were observed for untreated tumour spheres.

At this point, to determine cell viability, tumour spheres were released from the drug. Under these conditions, the growth capacity of spheroids was restored and shNT regrew up to reach approximately their original size (95.7%) on day 12 without pazopanib. In contrast, shISG15 spheres not only did not enlarge but also continued shrinking to 34.9% their original size (Fig. 5E, Supplementary Fig. 4). Similar effects were observed when spheroids were treated with trabectedin or doxorubicin, as drug removal resulted in the maintenance of the growth capacity only in the control group (Fig. 5E; Supplementary Fig. 4). By day 20 without trabectedin, shNT spheres did not show any further shrinking (60.6% their original size) while the size of ISG15-silenced spheres dropped to 33.1% (Fig. 5D, Supplementary Fig. 4). The same pattern was observed for doxorubicin (shNT remained essentially at 57.4% of the original size; shISG15 dropped to 30.5% (Fig. 5E, Supplementary Fig. 4). IEC139 tumourspheres were also released from drug exposure after 72 h, and their size was followed for 8 days post-treatment. At this point spheroids were not able to regrow, but IEC139 shISG15 spheroids were smaller relative to their original size than shNT: 29.5% vs 26.6% for trabectedin and 38.3% vs 31.6% for doxorubicin respectively. No significant differences were observed for pazopanib post-treatment (Fig. 5E).

## Discussion

The findings presented here validate the prognostic value of ISG15 in M-SFT [6] and the predictive relevance in the context of antiangiogenic treatment. These effects could be related to the important role of ISG15 in drug resistance and maintenance of CSC characteristics, as we observed in our preclinical models of this specific STS type.

ISG15 overexpression significantly correlated with worse OS, in our complete series (T-SFT and M-SFT), a result that was strongly dependent on the expression of this gene in M-SFT. Accordingly, in M-SFT patients, PFS was lower in patients with high *ISG15* gene expression. In contrast, in T-SFT ISG15 did not show an impact in survivals. In concordance with gene expression, both high ISG15 protein expression and strong ISG15 protein immunostaining correlated with lower OS, in the M-SFT cohort. However, when taking T-SFT patients into account, ISG15 at protein level loses its significance. The latter, together with *ISG15* gene expression being more robust in M-SFT, points at ISG15 as a prognostic marker in more advanced/malignant stages of this disease. Also, a positive statistical correlation is proven between *ISG15* expression and number of mitoses, which is a sign of malignancy. Several other studies based on clinical data have associated high ISG15 expression with unfavourable prognosis in cancer patients [19, 23, 29, 34], as well as with higher histological grade, tumour size or invasiveness [24]. Furthermore, in pancreatic adenocarcinoma, peripheral blood ISG15 has also been validated as a potential diagnostic biomarker of cancer patients when compared to healthy controls [35].

As it is well known, the expression of SFT markers STAT6, CD34, CD99 and bcl-2 can be lost in most aggressive tumours, following a process of dedifferentiation [36–39]. Thus, ISG15 being elevated in more aggressive SFT entities may imply its involvement in dedifferentiation and stemness enhancement. However, no association between SFT markers and ISG15/ISGylation has been described to date.

In our experimental conditions*,* ISG15 gene silencing inhibited proliferation, but not migration or invasion; in contrast with other published work that observed a decrease of either of these tumoral characteristics in different malignancies [22, 23, 25, 40, 41]. However, this might indicate that ISG15 pro-tumoral functions are heterogeneous and depend on the cancer type. On the other hand, ISG15 knockdown decreased the expression of specific markers associated with a CSC-like phenotype in our SFT cell line. Lower levels of sarcoma CSC markers *SOX2, NANOG, ALDH1A1, ABCB1 and ABCC1* [42] were observed in silenced cells in 2D and in CSC-enriched cultures (single-cell and 3D/spheroid).

Additionally, ISG15 induction shows a direct effect in the expression of the mentioned CSC-related genes. This is demonstrated by the increase in the expression of *SOX2, NANOG, ALDH1A1, ABCB1 and ABCC1* when ISG15 is induced by IFN-β or by drug treatment, which is not observed in ISG15-deprived silenced INT-SFT cells. Moreover, a greater ISG15 induction, as seen in trabectedin treatment, seems to correspond to a sharper increase in most of said CSC markers. Also, this effect is not replicated in IEC139 cells, which ISG15 silencing is not considerable enough to prevent its induction, and thus CSC markers upregulation. To our knowledge, it is the first time a positive correlation has been described between ISG15 and these CSC-related factors. The upregulation of CSC markers when treated with pazopanib, trabectedin or doxorubicin might warn us about the possible early activation of ISG15-regulated resistance mechanisms in SFT. Besides, some authors advise against the use of doxorubicin in SFT, at least as first-line, due to the addition of genomic instability [43]. Monitoring of ISG15 levels in SFT patients, throughout antiangiogenics or chemotherapy treatment, is needed to prove this hypothesis.

Furthermore, shISG15 cells presented lower tumour-initiating capacity, reflected by impaired spheroid forming ability and clonogenic capacity. In addition, cells expressing higher levels of ISG15 are more resistant to antiangiogenic pazopanib and chemotherapy drugs (doxorubicin and trabectedin) in 2D and 3D cultures; and are more capable of “reforming” spheroids after being damaged by drug administration. All these characteristics have been associated with a CSC phenotype. In general, CSC marker expression, colony/sphere-formation abilities and drug resistance are more reduced in INT-SFT than in IEC139 ISG15 knockdown. This corresponds with a greater ISG15 silencing in INT-SFT shISG15 cells, and might indicate that the CSC characteristics tested here could be proportionally dependent on ISG15 levels. Supporting our findings, other studies claimed that ISG15 promotes CSC behaviour, colony-forming capacity and tumorigenesis in nasopharyngeal and ductal pancreatic cancer cell lines [27–30, 44], which translates into a worse prognosis in these patients. Besides, ISG15 has been described to play a pro-tumoral role in various malignancies like bladder, nasopharyngeal, breast, hepatocellular or pancreatic cancer [18–24, 27–30, 44]. In sarcoma, a recent bioinformatics study into Ewing sarcoma, comparing tumour samples versus non-cancerous samples, identified ISG15 as one of the hub genes in a protein–protein interaction network (PPI) [45]. Conversely, ISG15 showed a protective anti-tumour role in glioblastoma and ovarian models [46, 47]. Because of these apparently contradictory functions, ISG15 has been designated as a “double-edged sword” in tumour development [48]; however, our data suggest that ISG15 has a pro-tumoral role in SFT.

ISG15 oncogenic mechanisms are also diverse, as it can act intracellularly in its free [27, 28] or its conjugated form [22, 25, 44]; as well as extracellularly as a microenvironmental modulator [23, 28, 30]. More specifically, both paracrine secretion from M2 macrophages and autocrine ISG15 secretion from pancreatic tumour cells are able to induce a CSC phenotype in said cells [28, 30]. However, the receptor binding to ISG15 or a possible positive correlation between secreted and intracellular/conjugated forms remains unknown. Our data support that autocrine tumour-secreted ISG15 may also play a role in CSC enhancement in an SFT context. Indeed, one of the limitations of this study is precisely related to the multiple physiological forms in which ISG15 can be found: secreted, intracellularly free and conjugated; which cannot be differentiated in patient samples at protein (IHC) or RNA level. Our knockdown experiments showed that all these forms of ISG15 are downregulated in INT-SFT, but ISG15 conjugates are not significantly reduced in IEC139, which could point that free ISG15 plays a more important role. Besides, transcriptomic analysis of ISG15 knockdown in SFT cells showed the downregulation of genes related to key CSC pathways like EMT, TGF-β or p53. However, how each one of these physiological forms can participate in SFT prognosis and the exact mechanism of action needs further research in preclinical models.

Overall, our in vitro studies suggest that ISG15 may be related to poor prognosis in SFT patients due to an enhancement in CSC phenotype. Thus, ISG15 presenting greater prognostic value in M-SFT patients may indicate its involvement in dedifferentiation and stemness processes and might function at latter stages of this tumour development. Moreover, ISG15-targets when ISGylated may vary their function; they can be degraded or protected from degradation at proteasome level, which leaves us with a rather complex situation. The role of ISGylation in SFT and other sarcomas is currently being studied in our laboratory. For future directions, a potential clinical trial could be designed considering ISG15 effect for advanced SFT patients. It would be of great interest to select patients with low ISG15 expression, expecting to improve clinical outcome when treated with efficiency-proven drugs like pazopanib.

## Conclusions

ISG15 is validated as a prognostic biomarker in M-SFT patients and could also present predictive value in antiangiogenic-treated patients. Our preclinical results suggest that worse prognosis could be a consequence of ISG15-mediated CSC behaviour and drug resistance mechanisms. These findings provide key information for future SFT clinical trials and a novel therapeutic target in this malignancy.

## Supplementary Information

Below is the link to the electronic supplementary material.

Supplementary figure 1. T-SFT cohort survival analysis by ISG15 levels, sarcoma vs normal tissue ISG15 expression and TMA ISG15 IHC. A) RNA B) Protein extension C) Protein intensity. D) SV40 LargeT antigen transformation does not produce ISG15 upregulation in HEK293 cells. E) R2 online tool comparing ISG15 levels between sarcoma and normal tissue datasets. F) Representative images of TMA ISG15 IHC for strong, low and negative intensity staining (left to right). Positive cases present nuclear and/or cytoplasmic localization. G) IF microscopy images showing endoplasmic reticulum (ER) in green, ISG15 in red and overlapping images in M-SFT cells. Images correspond to maximum projection of Z-stack, acquired using confocal microscopy. ISG15 does not seem to colocalize with ER (n = 3)

Supplementary figure 2. Proliferation and migration assays, colony-forming cultures and secreted ISG15 for transduced SFT cells. ISG15 drug-mediated induction for IEC139 CSC genes. A) INT-SFT cells were cultured in 10mm plates in FBS-free media, until confluency (48h). Then, media was collected and proteins were precipitated by DOC/TCA method. Proteins were resuspended in water and used for ISG15 WB. Ponceu S staining was used as loading control (n=2). B) Representative images of clonogenic cultures of transduced INT-SFT and IEC139 cells. ShISG15 present lower ability of colony formation (n=5). C) Migration was determined by wound healing assay. Pictures taken at 0, 6, 12 and 24h show no differences in migration capacity for shNT and shISG15. Graph represents the change in area of the wound over time (n=4). E) MYC gene expression shows significant downregulation in shISG15#2 3D cultures (n=3). F) IEC139 ISG15 knockdown is not considerable enough to prevent ISG15 induction when treated with 48h pazopanib, trabectedin or doxorubicin. Thus, CSC markers like SOX2 are also upregulated. (n=4) G) Gene set enrichment analysis (GSEA) showing CSC-related pathways affected by ISG15 silencing. T-student tests: (*) stands for p-value < 0.05. Independent experiments were performed

Supplementary Figure 3. Population of cells for Annexin V-FITC /PI apoptosis assay

Supplementary figure 4. ISG15 knockdown tumor spheres are not able to regenerate after toxic treatment. A) 72h 20 μM pazopanib B) 72h 0.5 nM trabectedin C) 50 nM doxorubicin D) Relative spheroid area to day 0 for each condition (n = 4). Spheroids were released from each drug at 72h, then images where obtained every day using an inverted microscope

## Data Availability

Not applicable.

## Abbreviations

SFT: Solitary fibrous tumour
T-SFT: Typical solitary fibrous tumour
M-SFT: Malignant solitary fibrous tumour
ISG15: Interferon-stimulated gene 15
CSC: Cancer stem cell
PFS: Progression-free survival
OS: Overall survival
shNT: Non-targeting short hairpin RNA
IHC: Immunohistochemistry

## References

[1] WHO (2020) Classification of Tumours Editorial B: *Soft tissue and bone tumours*. WHO

[2] Chmielecki J, Crago AM, Rosenberg M, O'Connor R, Walker SR, Ambrogio L, Auclair D, McKenna A, Heinrich MC, Frank DA, Meyerson M (2013). Whole-exome sequencing identifies a recurrent NAB2-STAT6 fusion in solitary fibrous tumors. Nat Genet.

[3] Robinson DR, Wu YM, Kalyana-Sundaram S, Cao X, Lonigro RJ, Sung YS, Chen CL, Zhang L, Wang R, Su F (2013). Identification of recurrent NAB2-STAT6 gene fusions in solitary fibrous tumor by integrative sequencing. Nat Genet.

[4] Mohajeri A, Tayebwa J, Collin A, Nilsson J, Magnusson L, von Steyern FV, Brosjö O, Domanski HA, Larsson O, Sciot R (2013). Comprehensive genetic analysis identifies a pathognomonic NAB2/STAT6 fusion gene, nonrandom secondary genomic imbalances, and a characteristic gene expression profile in solitary fibrous tumor. Genes Chromosomes Cancer.

[5] Fletcher CDM, International Agency for Research on C, Organización Mundial de la S (2013). WHO classification of tumours of soft tissue and bone.

[6] Martin-Broto J, Stacchiotti S, Lopez-Pousa A, Redondo A, Bernabeu D, de Alava E, Casali PG, Italiano A, Gutierrez A, Moura DS (2019). Pazopanib for treatment of advanced malignant and dedifferentiated solitary fibrous tumour: a multicentre, single-arm, phase 2 trial. Lancet Oncol.

[7] Martin-Broto J, Cruz J, Penel N, Le Cesne A, Hindi N, Luna P, Moura DS, Bernabeu D, de Alava E, Lopez-Guerrero JA (2020). Pazopanib for treatment of typical solitary fibrous tumours: a multicentre, single-arm, phase 2 trial. Lancet Oncol.

[8] Korant BD, Blomstrom DC, Jonak GJ, Knight E (1984). Interferon-induced proteins. Purification and characterization of a 15,000-dalton protein from human and bovine cells induced by interferon. J Biol Chem.

[9] Haas AL, Ahrens P, Bright PM, Ankel H (1987). Interferon induces a 15-kilodalton protein exhibiting marked homology to ubiquitin. J Biol Chem.

[10] Loeb KR, Haas AL (1992). The interferon-inducible 15-kDa ubiquitin homolog conjugates to intracellular proteins. J Biol Chem.

[11] Der SD, Zhou A, Williams BR, Silverman RH (1998). Identification of genes differentially regulated by interferon alpha, beta, or gamma using oligonucleotide arrays. Proc Natl Acad Sci USA.

[12] Yuan W, Krug RM (2001). Influenza B virus NS1 protein inhibits conjugation of the interferon (IFN)-induced ubiquitin-like ISG15 protein. Embo j.

[13] Liu M, Hummer BT, Li X, Hassel BA (2004). Camptothecin induces the ubiquitin-like protein, ISG15, and enhances ISG15 conjugation in response to interferon. J Interferon Cytokine Res.

[14] Park JH, Yang SW, Park JM, Ka SH, Kim JH, Kong YY, Jeon YJ, Seol JH, Chung CH (2016). Positive feedback regulation of p53 transactivity by DNA damage-induced ISG15 modification. Nat Commun.

[15] Jeon YJ, Park JH, Chung CH (2017). Interferon-stimulated gene 15 in the control of cellular responses to genotoxic stress. Mol Cells.

[16] Khaminets A, Behl C, Dikic I (2016). Ubiquitin-dependent and independent signals in selective autophagy. Trends Cell Biol.

[17] Bhushan J, Radke JB, Perng YC, McAllaster M, Lenschow DJ, Virgin HW, Sibley LD (2020). ISG15 connects autophagy and IFN-γ-Dependent control of Toxoplasma gondii infection in human cells. mBio.

[18] Andersen JB, Aaboe M, Borden EC, Goloubeva OG, Hassel BA, Orntoft TF (2006). Stage-associated overexpression of the ubiquitin-like protein, ISG15, in bladder cancer. Br J Cancer.

[19] Bektas N, Noetzel E, Veeck J, Press MF, Kristiansen G, Naami A, Hartmann A, Dimmler A, Beckmann MW, Knüchel R (2008). The ubiquitin-like molecule interferon-stimulated gene 15 (ISG15) is a potential prognostic marker in human breast cancer. Breast Cancer Res.

[20] Desai SD, Reed RE, Burks J, Wood LM, Pullikuth AK, Haas AL, Liu LF, Breslin JW, Meiners S, Sankar S (2012). ISG15 disrupts cytoskeletal architecture and promotes motility in human breast cancer cells. Exp Biol Med (Maywood).

[21] Laljee RP, Muddaiah S, Salagundi B, Cariappa PM, Indra AS, Sanjay V, Ramanathan A (2013). Interferon stimulated gene-ISG15 is a potential diagnostic biomarker in oral squamous cell carcinomas. Asian Pac J Cancer Prev.

[22] Burks J, Reed RE, Desai SD (2014). ISGylation governs the oncogenic function of Ki-Ras in breast cancer. Oncogene.

[23] Li C, Wang J, Zhang H, Zhu M, Chen F, Hu Y, Liu H, Zhu H (2014). Interferon-stimulated gene 15 (ISG15) is a trigger for tumorigenesis and metastasis of hepatocellular carcinoma. Oncotarget.

[24] Kariri YA, Alsaleem M, Joseph C, Alsaeed S, Aljohani A, Shiino S, Mohammed OJ, Toss MS, Green AR, Rakha EA (2021). The prognostic significance of interferon-stimulated gene 15 (ISG15) in invasive breast cancer. Breast Cancer Res Treat.

[25] Bolado-Carrancio A, Lee M, Ewing A, Muir M, Macleod KG, Gallagher WM, Nguyen LK, Carragher NO, Semple CA, Brunton VG (2021). ISGylation drives basal breast tumour progression by promoting EGFR recycling and Akt signalling. Oncogene.

[26] Li YL, Gao YL, Niu XL, Wu YT, Du YM, Tang MS, Li JY, Guan XH, Song B (2020). Identification of subtype-specific metastasis-related genetic signatures in sarcoma. Front Oncol.

[27] Li XY, Yan J, Sun J, Li C, Jiang JY, Wang JM, Meng XN, Liang JJ, Wang HQ (2019). BAG3 deletion suppresses stem cell-like features of pancreatic ductal adenocarcinoma via translational suppression of ISG15. Biochim Biophys Acta Mol Cell Res.

[28] Sun J, Yan J, Qiao HY, Zhao FY, Li C, Jiang JY, Liu BQ, Meng XN, Wang HQ (2020). Loss of TRIM29 suppresses cancer stem cell-like characteristics of PDACs via accelerating ISG15 degradation. Oncogene.

[29] Chen RH, Du Y, Han P, Wang HB, Liang FY, Feng GK, Zhou AJ, Cai MY, Zhong Q, Zeng MS, Huang XM (2016). ISG15 predicts poor prognosis and promotes cancer stem cell phenotype in nasopharyngeal carcinoma. Oncotarget.

[30] Sainz B, Martín B, Tatari M, Heeschen C, Guerra S (2014). ISG15 is a critical microenvironmental factor for pancreatic cancer stem cells. Cancer Res.

[31] Spagnuolo RD, Brich S, Bozzi F, Conca E, Castelli C, Tazzari M, Maestro R, Brenca M, Gualeni AV, Gloghini A (2016). Sunitinib-induced morpho-functional changes and drug effectiveness in malignant solitary fibrous tumours. Oncotarget.

[32] Kessel S, Cribbes S, Déry O, Kuksin D, Sincoff E, Qiu J, Chan LL (2017). High-throughput 3D tumor spheroid screening method for cancer drug discovery using celigo image cytometry. SLAS Technol.

[33] Sirenko O, Mitlo T, Hesley J, Luke S, Owens W, Cromwell EF (2015). High-content assays for characterizing the viability and morphology of 3D cancer spheroid cultures. Assay Drug Dev Technol.

[34] Wan B, Liu B, Huang Y, Yu G, Lv C (2019). Prognostic value of immune-related genes in clear cell renal cell carcinoma. Aging (Albany NY).

[35] Zuo D, Chen Y, Zhang X, Wang Z, Jiang W, Tang F, Cheng R, Sun Y, Sun L, Ren L, Liu R (2021) Identification of hub genes and their novel diagnostic and prognostic significance in pancreatic adenocarcinoma. Cancer Biol Med 10.20892/j.issn.2095-3941.2020.0516 PMC9334760 34403221

[36] Yokoi T, Tsuzuki T, Yatabe Y, Suzuki M, Kurumaya H, Koshikawa T, Kuhara H, Kuroda M, Nakamura N, Nakatani Y, Kakudo K (1998). Solitary fibrous tumour: significance of p53 and CD34 immunoreactivity in its malignant transformation. Histopathology.

[37] Ouladan S, Trautmann M, Orouji E, Hartmann W, Huss S, Büttner R, Wardelmann E (2015). Differential diagnosis of solitary fibrous tumors: A study of 454 soft tissue tumors indicating the diagnostic value of nuclear STAT6 relocation and ALDH1 expression combined with in situ proximity ligation assay. Int J Oncol.

[38] Han Y, Zhang Q, Yu X, Han X, Wang H, Xu Y, Qiu X, Jin F (2015). Immunohistochemical detection of STAT6, CD34, CD99 and BCL-2 for diagnosing solitary fibrous tumors/hemangiopericytomas. Int J Clin Exp Pathol.

[39] Schneider N, Hallin M, Thway K (2017). STAT6 loss in dedifferentiated solitary fibrous tumor. Int J Surg Pathol.

[40] Kong E, Kim HD, Kim J (2020). Deleting key autophagy elongation proteins induces acquirement of tumor-associated phenotypes via ISG15. Cell Death Differ.

[41] Cheriyamundath S, Basu S, Haase G, Doernberg H, Gavert N, Brabletz T, Ben-Ze'ev A (2019). ISG15 induction is required during L1-mediated colon cancer progression and metastasis. Oncotarget.

[42] Martínez-Delgado P, Lacerenza S, Obrador-Hevia A, Lopez-Alvarez M, Mondaza-Hernandez JL, Blanco-Alcaina E, Sanchez-Bustos P, Hindi N, Moura DS, Martin-Broto J (2020). Cancer stem cells in soft-tissue sarcomas. Cells.

[43] Martin-Broto J, Mondaza-Hernandez JL, Moura DS, Hindi N (2021). A comprehensive review on solitary fibrous tumor: new insights for new horizons. Cancers (Basel).

[44] Alcalá S, Sancho P, Martinelli P, Navarro D, Pedrero C, Martín-Hijano L, Valle S, Earl J, Rodríguez-Serrano M, Ruiz-Cañas L (2020). ISG15 and ISGylation is required for pancreatic cancer stem cell mitophagy and metabolic plasticity. Nat Commun.

[45] Zhang J, Zhang Y, Li Z, Wu H, Xun J, Feng H (2019). Bioinformatics analysis of Ewing's sarcoma: Seeking key candidate genes and pathways. Oncol Lett.

[46] Du Z, Cai C, Sims M, Boop FA, Davidoff AM, Pfeffer LM (2017). The effects of type I interferon on glioblastoma cancer stem cells. Biochem Biophys Res Commun.

[47] Zhang Q, Wang J, Qiao H, Huyan L, Liu B, Li C, Jiang J, Zhao F, Wang H, Yan J (2021). ISG15 is downregulated by KLF12 and implicated in maintenance of cancer stem cell-like features in cisplatin-resistant ovarian cancer. J Cell Mol Med.

[48] Desai SD (2015). ISG15: a double edged sword in cancer. Oncoimmunology.

