# Supplementary figures and images for: ISG15 as a prognostic biomarker in solitary fibrous tumour
Source: Cell Mol Life Sci. 2022 Jul 21;79(8):434. doi: 10.1007/s00018-022-04454-4 (PMC9304060; doi:10.1007/s00018-022-04454-4)

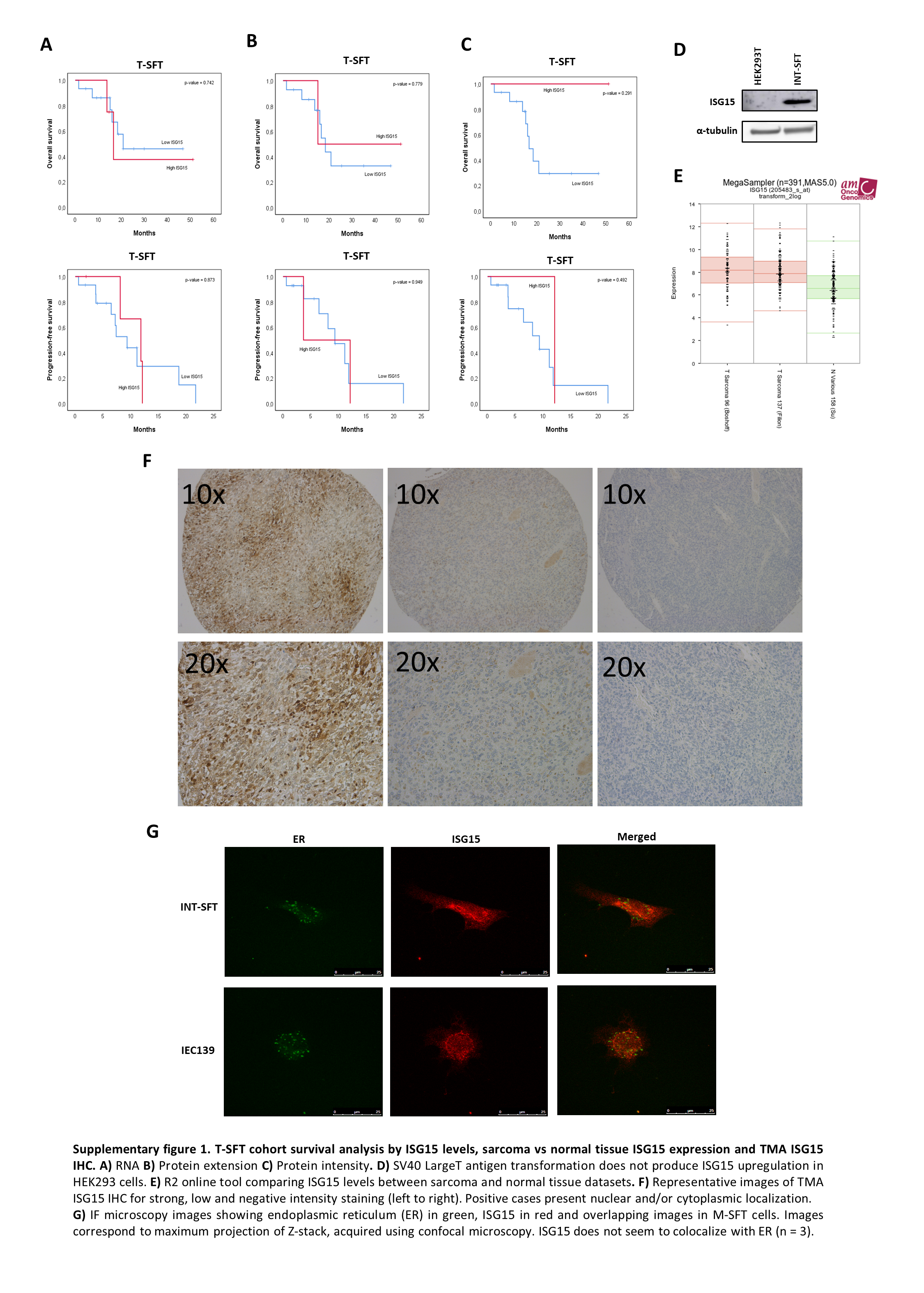

Supplement: Supplementary file 1 — Supplementary figure 1. T-SFT cohort survival analysis by ISG15 levels, sarcoma vs normal tissue ISG15 expression and TMA ISG15 IHC. A) RNA B) Protein extension C) Protein intensity. D) SV40 LargeT antigen transformation does not produce ISG15 upregulation in HEK293 cells. E) R2 online tool comparing ISG15 levels between sarcoma and normal tissue datasets. F) Representative images of TMA ISG15 IHC for strong, low and negative intensity staining (left to right). Positive cases present nuclear and/or cytoplasmic localization. G) IF microscopy images showing endoplasmic reticulum (ER) in green, ISG15 in red and overlapping images in M-SFT cells. Images correspond to maximum projection of Z-stack, acquired using confocal microscopy. ISG15 does not seem to colocalize with ER (n = 3) [file 18_2022_4454_MOESM1_ESM.tif]

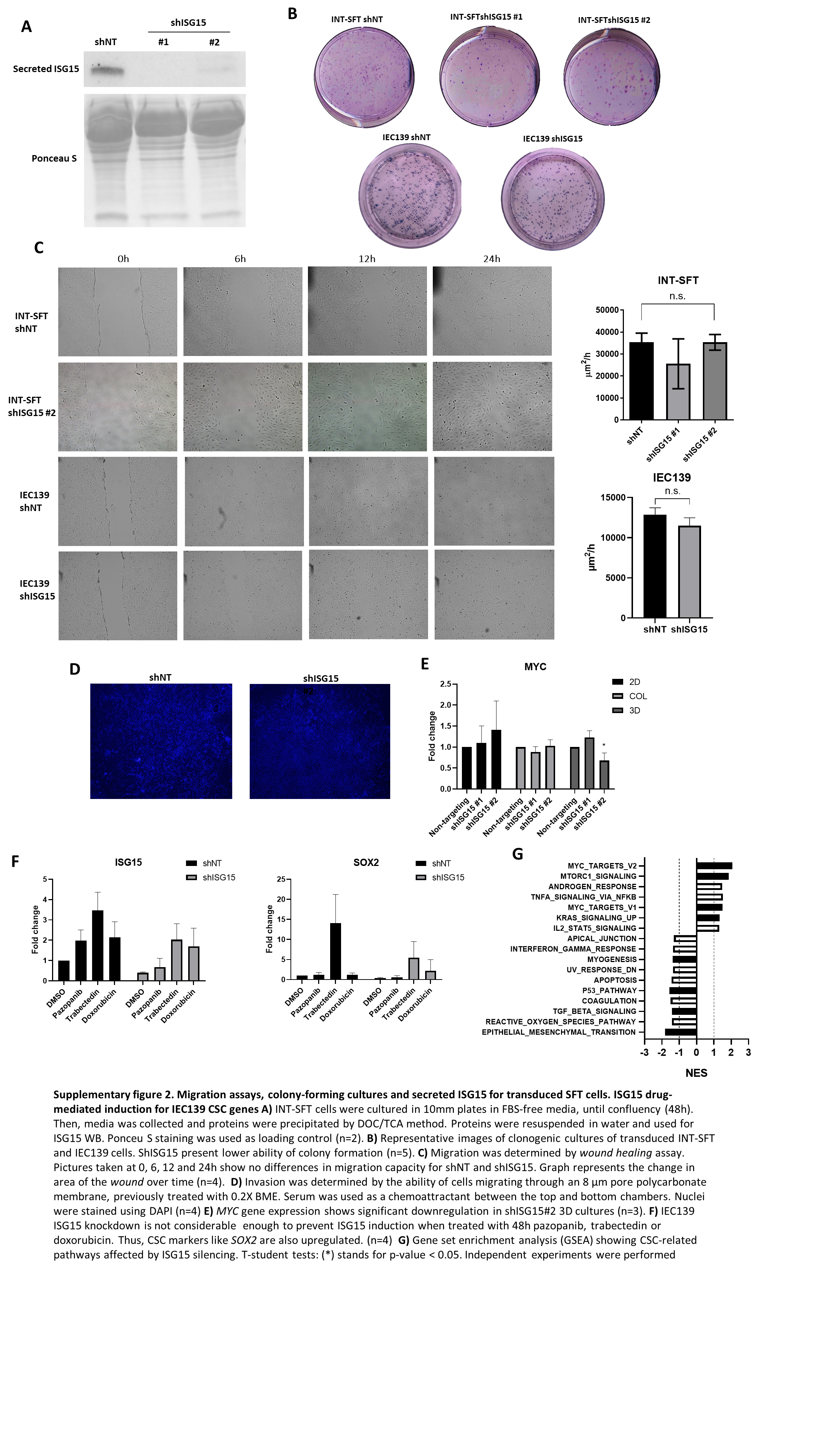

Supplement: Supplementary file 2 — Supplementary figure 2. Proliferation and migration assays, colony-forming cultures and secreted ISG15 for transduced SFT cells. ISG15 drug-mediated induction for IEC139 CSC genes. A) INT-SFT cells were cultured in 10mm plates in FBS-free media, until confluency (48h). Then, media was collected and proteins were precipitated by DOC/TCA method. Proteins were resuspended in water and used for ISG15 WB. Ponceu S staining was used as loading control (n=2). B) Representative images of clonogenic cultures of transduced INT-SFT and IEC139 cells. ShISG15 present lower ability of colony formation (n=5). C) Migration was determined by wound healing assay. Pictures taken at 0, 6, 12 and 24h show no differences in migration capacity for shNT and shISG15. Graph represents the change in area of the wound over time (n=4). E) MYC gene expression shows significant downregulation in shISG15#2 3D cultures (n=3). F) IEC139 ISG15 knockdown is not considerable enough to prevent ISG15 induction when treated with 48h pazopanib, trabectedin or doxorubicin. Thus, CSC markers like SOX2 are also upregulated. (n=4) G) Gene set enrichment analysis (GSEA) showing CSC-related pathways affected by ISG15 silencing. T-student tests: (*) stands for p-value < 0.05. Independent experiments were performed [file 18_2022_4454_MOESM2_ESM.tif]

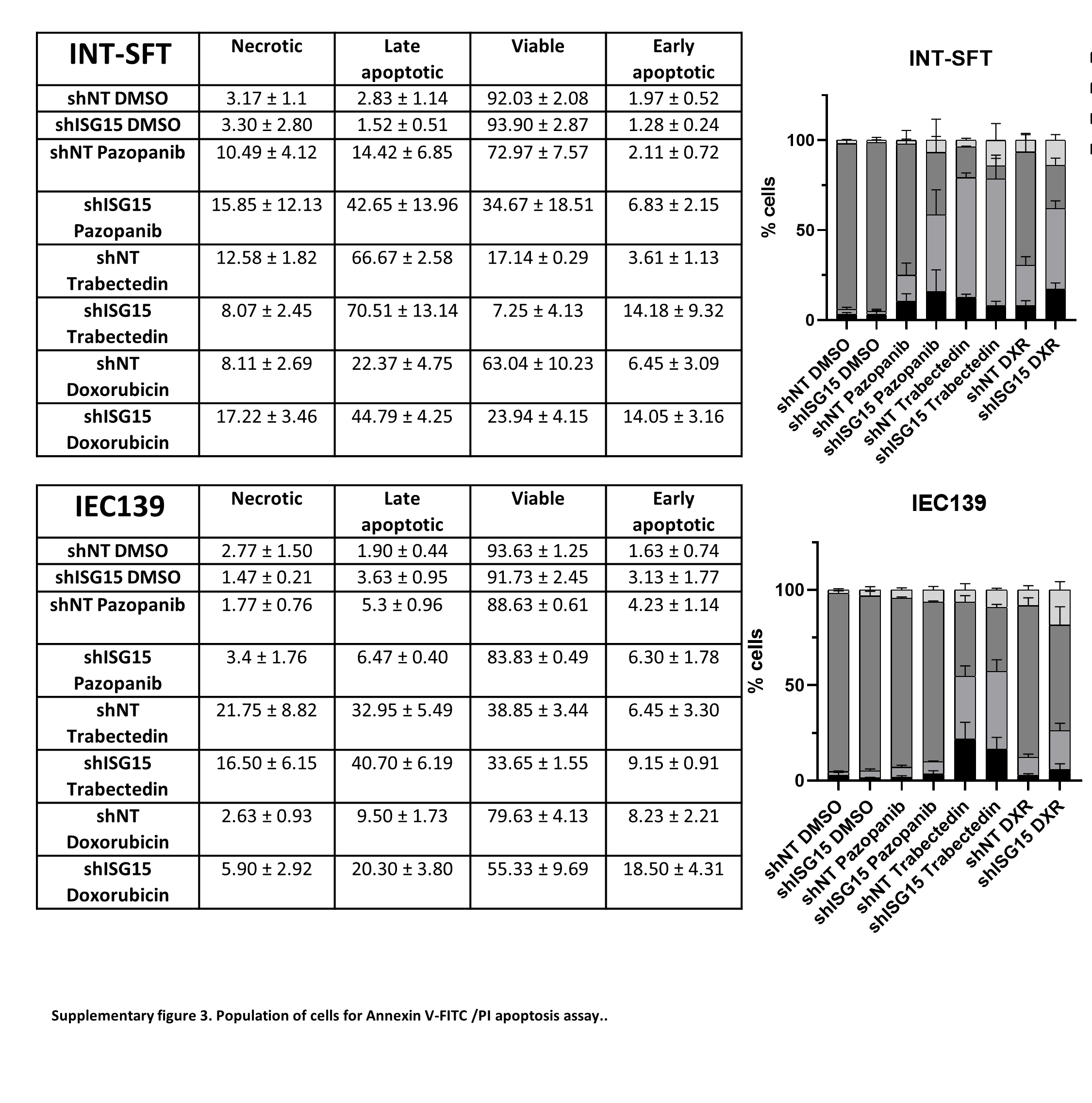

Supplement: Supplementary file 3 — Supplementary Figure 3. Population of cells for Annexin V-FITC /PI apoptosis assay [file 18_2022_4454_MOESM3_ESM.tif]

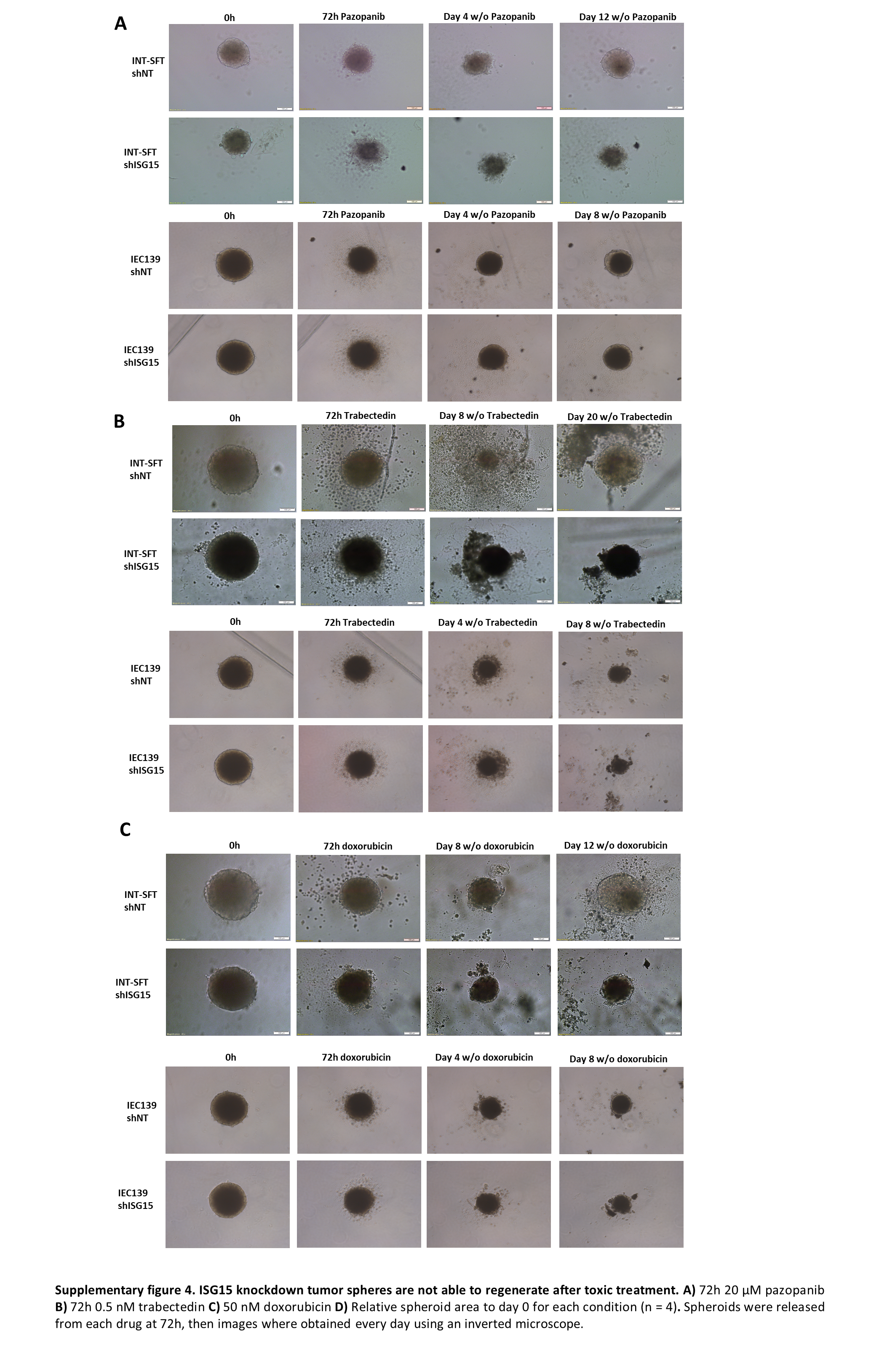

Supplement: Supplementary file 4 — Supplementary figure 4. ISG15 knockdown tumor spheres are not able to regenerate after toxic treatment. A) 72h 20 μM pazopanib B) 72h 0.5 nM trabectedin C) 50 nM doxorubicin D) Relative spheroid area to day 0 for each condition (n = 4). Spheroids were released from each drug at 72h, then images where obtained every day using an inverted microscope [file 18_2022_4454_MOESM4_ESM.tif]
